# Olfactory Stem Cells, a New Cellular Model for Studying Molecular Mechanisms Underlying Familial Dysautonomia

**DOI:** 10.1371/journal.pone.0015590

**Published:** 2010-12-20

**Authors:** Nathalie Boone, Béatrice Loriod, Aurélie Bergon, Oualid Sbai, Christine Formisano-Tréziny, Jean Gabert, Michel Khrestchatisky, Catherine Nguyen, François Féron, Felicia B. Axelrod, El Chérif Ibrahim

**Affiliations:** 1 NICN-CNRS UMR 6184, Université de la Méditerranée-Faculté de Médecine Nord, IFR Jean Roche, Marseille, France; 2 TAGC, INSERM U928, Marseille, France; 3 Plateforme Transcriptome, CRO2, Faculté de Médecine, Marseille, France; 4 Biochemistry and Molecular Biology, Hôpital Nord, AP-HM, Marseille, France; 5 Department of Pediatrics, New York University School of Medicine, New York, New York, United States of America; Istituto Dermopatico dell'Immacolata, Italy

## Abstract

**Background:**

Familial dysautonomia (FD) is a hereditary neuropathy caused by mutations in the *IKBKAP* gene, the most common of which results in variable tissue-specific mRNA splicing with skipping of exon 20. Defective splicing is especially severe in nervous tissue, leading to incomplete development and progressive degeneration of sensory and autonomic neurons. The specificity of neuron loss in FD is poorly understood due to the lack of an appropriate model system. To better understand and modelize the molecular mechanisms of *IKBKAP* mRNA splicing, we collected human olfactory ecto-mesenchymal stem cells (hOE-MSC) from FD patients. hOE-MSCs have a pluripotent ability to differentiate into various cell lineages, including neurons and glial cells.

**Methodology/Principal Findings:**

We confirmed *IKBKAP* mRNA alternative splicing in FD hOE-MSCs and identified 2 novel spliced isoforms also present in control cells. We observed a significant lower expression of both *IKBKAP* transcript and IKAP/hELP1 protein in FD cells resulting from the degradation of the transcript isoform skipping exon 20. We localized IKAP/hELP1 in different cell compartments, including the nucleus, which supports multiple roles for that protein. We also investigated cellular pathways altered in FD, at the genome-wide level, and confirmed that cell migration and cytoskeleton reorganization were among the processes altered in FD. Indeed, FD hOE-MSCs exhibit impaired migration compared to control cells. Moreover, we showed that kinetin improved exon 20 inclusion and restores a normal level of IKAP/hELP1 in FD hOE-MSCs. Furthermore, we were able to modify the *IKBKAP* splicing ratio in FD hOE-MSCs, increasing or reducing the WT (exon 20 inclusion):MU (exon 20 skipping) ratio respectively, either by producing free-floating spheres, or by inducing cells into neural differentiation.

**Conclusions/Significance:**

hOE-MSCs isolated from FD patients represent a new approach for modeling FD to better understand genetic expression and possible therapeutic approaches. This model could also be applied to other neurological genetic diseases.

## Introduction

Familial dysautonomia (FD, Riley-Day syndrome, hereditary sensory and autonomic neuropathy type III, MIM 223900) is an autosomal recessive genetic disorder that occurs in 1∶3600 live births with a carrier frequency of 1 in 30 in the Ashkenazi Jewish population. The disease is characterized by incomplete development and the progressive depletion of autonomic and sensory neurons [1]–[3] resulting in variable symptoms including: insensitivity to pain, lack of overflow tearing, inappropriate blood pressure control manifested as orthostatic hypotension and episodic hypertension, poor oral coordination resulting in poor feeding and swallowing, and gastrointestinal dysmotility [4]. No cure is available for this disorder and treatment is aimed at controlling symptoms and avoiding complications.

FD is caused by mutations in the *IKBKAP* gene which encodes a protein termed IKAP/hELP1 [5], [6]. The most prevalent mutation, is a splice mutation; the T-to-C transition in position 6 of the 5′ splice site (5′ss) of intron 20 (IVS20^+6T→C^) of this gene. All FD cases have at least one copy of this mutation; >99.5% are homozygous [5]–[7]. This mutation leads to variable, tissue-specific skipping of exon 20 of *IKBKAP* mRNA, with the central and peripheral nervous system more prone to complete skipping than others tissues, which leads to reduced IKAP/hELP1 protein levels [8].

Although the exact function of the IKAP/hELP1 protein is not clearly understood, researchers have identified IKAP/hELP1 as the scaffold protein required to assemble a well conserved six-protein complex (ELP1-6) called the holo-Elongator complex that possess histone acetyltransferase activity directed against histone H3 and H4 *in vitro* [9]. IKAP/hElongator is recruited to the transcribed regions of some human genes essentially involved in actin cytoskeleton regulation and cell motility migration [10]. This role may underlie a cell motility deficiency in FD neurons because of impaired transcriptional elongation of some genes coding for proteins involved in cell migration. Indeed, one study found that mouse neurons defective in Elongator exhibit reduced levels of acetylated α-tubulin, causing defects in radial migration and branching of cortical projections neurons [11]. Another study showed that *Caenorhabditis elegans* Elongator complex is required for correct acetylation of microtubules and neuronal development [12]. IKAP/hELP1 protein is also involved in other cellular processes, including tRNA modifications [13]–[15], exocytosis [16], and zygotic paternal genome demethylation [17]. Recently, its homolog in fly (D-elp1) has also been suggested to be involved in RNA interference through a RNA-dependent RNA polymerase activity [18].

To better understand the molecular mechanisms leading to aberrant splicing of *IKBKAP* mRNA in FD, creation of model systems recapitulating the pathological development of neural cells is required. Because *IKBKAP* gene knock out causes embryonic lethality [19], an animal model that exhibits the major phenotypic characteristics observed in FD humans has not yet been established. However, a humanized *IKBKAP* transgenic mouse model for FD has been created [20], that reproduces the tissue-specific splicing of *IKBKAP* mRNA in nervous tissues. Such a model is a notable progress in the comprehension of this complex rare disease and offers a potential system for testing therapeutic agents. However, transgenic animals do not reproduce phenotypic features of FD as they maintain normal development. Alternatively, FD patient fibroblasts are an informative model of mRNA splicing regulation. However, a recent study suggests that IKAP/hELP1 expression is much higher in neurons compared to fibroblasts [21], and fibroblasts do not exhibit the same ratio of *IKBKAP* exon 20 including∶exon 20 skipping transcripts (named WT∶MU respectively for simplicity) as observed in nervous system-derived tissues [8]. This finding narrows the understanding of disease mechanisms in a neural context. Finally, generation of neural cells through the production of induced pluripotent stem (iPS) cell from FD fibroblasts has been recently established [22]. Neural cells derived from iPS cells have potential to be used for studies of neuropathologies [23]. However, the labor intensive reprogramming required to induce iPS cells erases the developmentally relevant epigenetic signature specific to the disease state. As a consequence, some important information may be lost impeding recreation of an accurate disease model. The demonstration that fibroblasts can be converted directly into neurons, without an initial reprogramming, as recently evidenced in mouse [24], is very attractive. Nevertheless, during their reprogramming, human iPS cells do not pass through the normal stages of embryonic development that human ES cells undergo. Although both stem cell types share a common transcriptional signature, a subset of genetic profiles found in human iPS cells suggests retention of transcriptional and epigenetic memory related to their tissue of origin, which can substantially affect their potential to differentiate into different cell types [25]–[27]. Thus, cells collected from primary sources that have been subjected to environmental signals appropriate for the pathological specificity of the targeted disease are likely important to mirror the biology of diseased human neural cells.

Our aim is to understand what mechanisms drive *IKBKAP* mRNA splicing to the almost exclusive production of aberrant transcripts (MU) in neuronal cells. Here we demonstrate the potential of human olfactory ecto-mesenchymal stem cells (hOE-MSCs) to model this aspect in FD. Indeed, neurogenesis occurs throughout adult life in the olfactory mucosa, due to the presence of resident multipotent stem cells giving rise to olfactory neurons *in vivo* [28]. hOE-MSCs can be grown into neurospheres, that are multipotent, and differentiate *in vitro* into neurons, astrocytes, and oligodendrocytes as well as other cell types [29], [30]. Isolated from patients, cultures of hOE-MSCs provide potential models for genetically determined neuropsychiatric diseases [31]–[33], and stand as an interesting human model to investigate gene networks and cellular pathways altered in disease like FD. For example, cell migration defects have been observed in cells lacking normal expression of IKAP/hELP1 [10], [11], [22], [34], and we show here that FD hOE-MSCs exhibit impaired migration compared to control cells. Additionally, hOE-MSCs are an appropriate model for validating the potency of therapeutic agents such as kinetin, a cytokinin that has been shown to increase *IKBKAP* mRNA and protein expression in FD cell lines and *in vivo* models [20], [22], [35], [36] as well as in leukocytes of healthy carriers of the FD mutation [37].

## Results

### FD hOE-MSCs express stem cell, glial and immature neuronal markers

**Figure 1:**
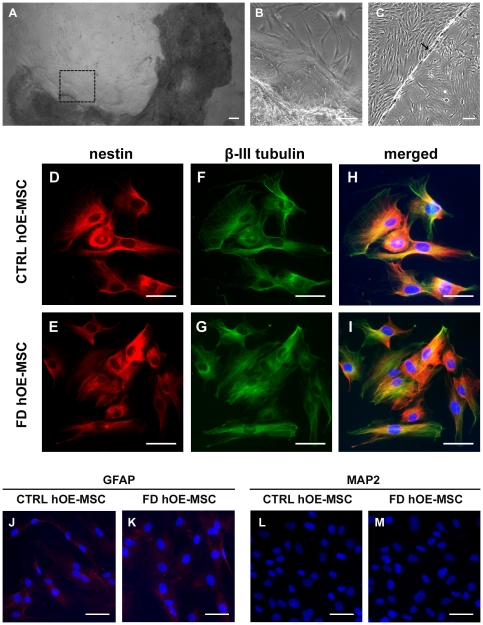
hOE-MSCs display characteristics of immature neuroglial cells. A, lamina propria (dark) from an FD olfactory mucosa biopsy was placed under a glass coverslip to initiate stem cell proliferation. Area delimited by a black square is enlarged in B. C, after transfer in a 6-well plate, cells attached to the coverslip (arrow) proliferated and colonized the complete area of the well. (D–M), Immunofluorescence stainings of both control (CTRL) and FD hOE-MSCs are positive for nestin (D, E), β-III tubulin (F,G) with similar expression levels (H, I) while slightly positive for GFAP (J,K) and negative for MAP2 (L, M). Green represents Alexa fluor-488, red Alexa Fluor-594. Nuclei (blue) were stained with Hoechst blue. Scale bars represent 50 µm.

To establish a human cellular model of FD, we collected 4 olfactory mucosa biopsies from homozygous patients for the IVS20^+6T→C^ FD mutation. As previously demonstrated with control biopsies [30], after about 2 weeks of culture, the microscopic examination of the tissue crushed under a glass coverslip revealed stem cell proliferation (Figure 1A and 1B). After reaching confluency in a 4-well plate, the cells attached to the glass coverslip were further expanded by transfer into a 6-well plate (Figure 1C). Like control hOE-MSCs, we observed that FD hOE-MSCs could be cultured for long periods (at least 15 cycles of trypsin/EDTA treatment and expansion on larger plastic surface) with a doubling time of about 30–48 h. When subjected to immunostaining, all hOE-MSCs derived from control and FD biopsies express the neural stem cell–specific marker nestin (Figure 1D and 1E) and the immature neuronal marker β-III tubulin (Figure 1F and 1G) in the same proportions (Figure 1H and 1I). A comparatively low GFAP staining was observed in every hOE-MSCs (Figure 1J and 1K). In addition, cells were negative for a mature neuronal marker, MAP2 (Figure 1L and 1M). This analysis suggests that both control and FD hOE-MSCs display properties of neuroglial progenitor cells.

### Expression of *IKBKAP* transcripts is dramatically reduced in FD hOE-MSCs

**Figure 2:**
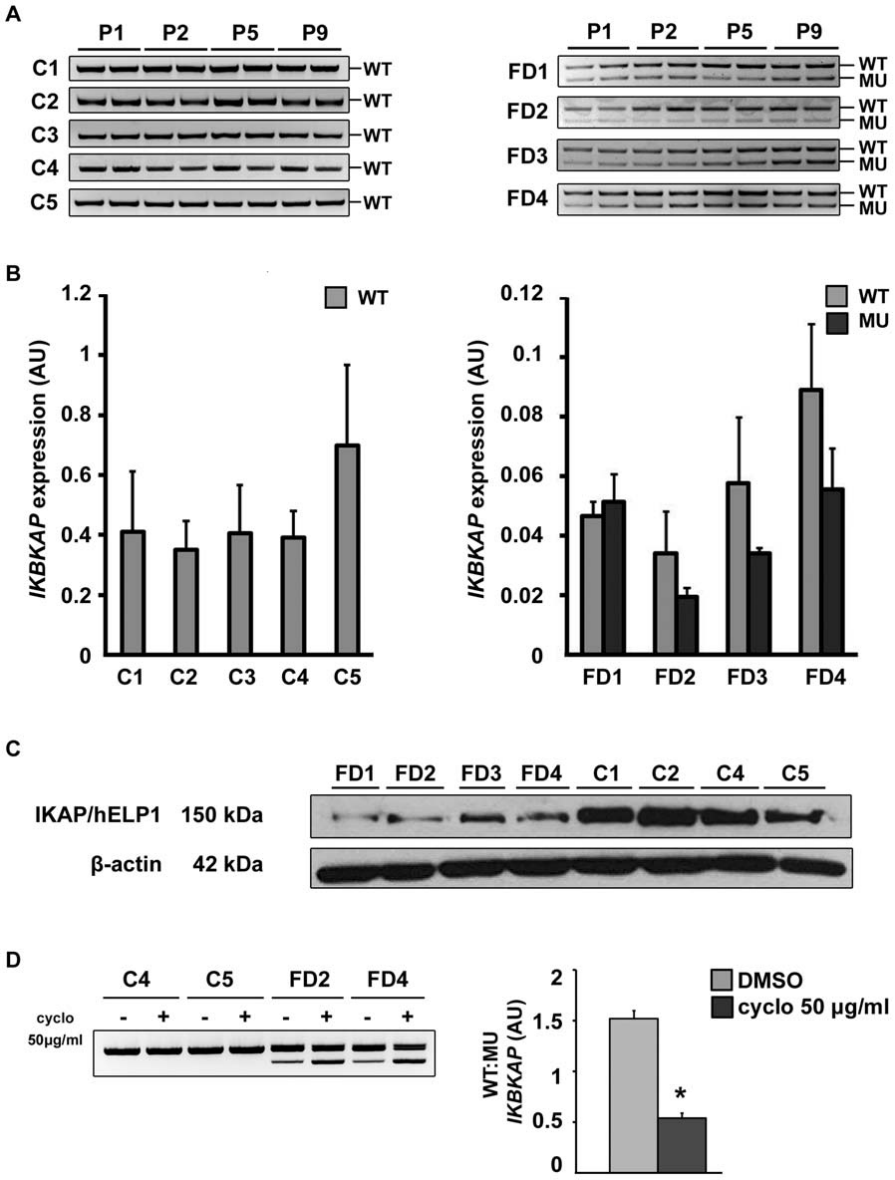
Expression of *IKBKAP* transcripts and IKAP/hELP1 protein in hOE-MSCs. A, agarose gel electrophoresis of end-point RT-PCR products showing both WT and MU transcripts of *IKBKAP* gene for control (left panel) and FD hOE-MSCs (right panel) at cell passage 1,2,5,9. B, graph of the mean level of expression of *IKBKAP* alternative transcripts in control (left panel) and FD hOE-MSCs (right panel) at cell passages 1,2,5,9, determined by absolute RT-qPCR. *ABL1* was used as a reference gene for normalization. Error barrs denote standard error. C, western blot analysis of total lysate from 4 controls and 4 FD hOE-MSCs using monoclonal anti-IKAP/hELP1 antibody (upper panel). Anti-β-actin was included to show equal loading (lower panel). D, NMD pathway was blocked by the translation inhibitor cycloheximide and results in an elevated expression of MU transcripts in FD cells (agarose gel electrophoresis, left panel). Results are confirmed with absolute qPCR normalized with *ABL1* (right panel).

*IKBKAP* mRNA expression was investigated in cultures of 5 controls and 4 FD hOE-MSCs at early (P1, P2) and later cell passages (P5, P9). A semi quantitative RT-PCR analysis revealed that, while control hOE-MSCs expressed exclusively the WT mRNA transcript (Figure 2A, left panel), FD hOE-MSCs expressed the WT but also the MU transcript (Figure 2A, right panel). We also demonstrated that long time culture conditions and trypsin-EDTA mediated cell passages did not affect the *IKBKAP* gene expression pattern. In order to more accurately determine the level of expression of *IKBKAP* alternative transcripts, we designed primers, probes and plasmid calibrators to perform absolute quantification using quantitative real-time RT-PCR (RT-qPCR) on the same samples. Strikingly, WT transcripts were much less expressed in FD (5–8 fold), when compared to controls hOE-MSCs (Figure 2B). In addition, WT and MU transcripts were present in nearly equal amounts in FD hOE-MSCs (Figure 2B, right graph). Furthermore, the total amount of *IKBKAP* transcripts in FD (WT+MU) remains 3 to 5 times less abundant than WT in controls, which suggests a defect in *IKBKAP* transcription and/or mRNA stability. In FD cells, the differential expression of *IKBKAP* transcripts was also correlated to a reduced expression of IKAP/hELP1 protein in FD, when compared to controls, as revealed by western blot analysis (Figure 2C). Since MU transcripts contain a premature stop codon that may activate the nonsense-mediated mRNA decay (NMD) pathway, we wanted to confirm whether this pathway is responsible for the lower *IKBKAP* transcripts expression in FD cells. Thus, we tested cycloheximide, a protein synthesis inhibitor which also inhibits NMD. Indeed, FD cells preincubated for 6 h with cycloheximide exhibited a stabilization of the MU transcript as evidenced by semi-quantitative RT-PCR (Figure 2D, left panel). To accurately determine the level of WT and MU *IKBKAP* transcripts in these samples, absolute RT-qPCR analysis was performed (Figure 2D, right panel). The results clearly demonstrated that the WT∶MU ratio decreases when mRNA surveillance is inhibited. Thus, a large amount of *IKBKAP* MU transcripts is degraded through the NMD pathway resulting in much less *IKBKAP* transcripts and IKAP/hELP1 protein in FD compared to control cells.

### Heterogeneous IKAP/hELP1 distribution in hOE-MSCs

**Figure 3:**
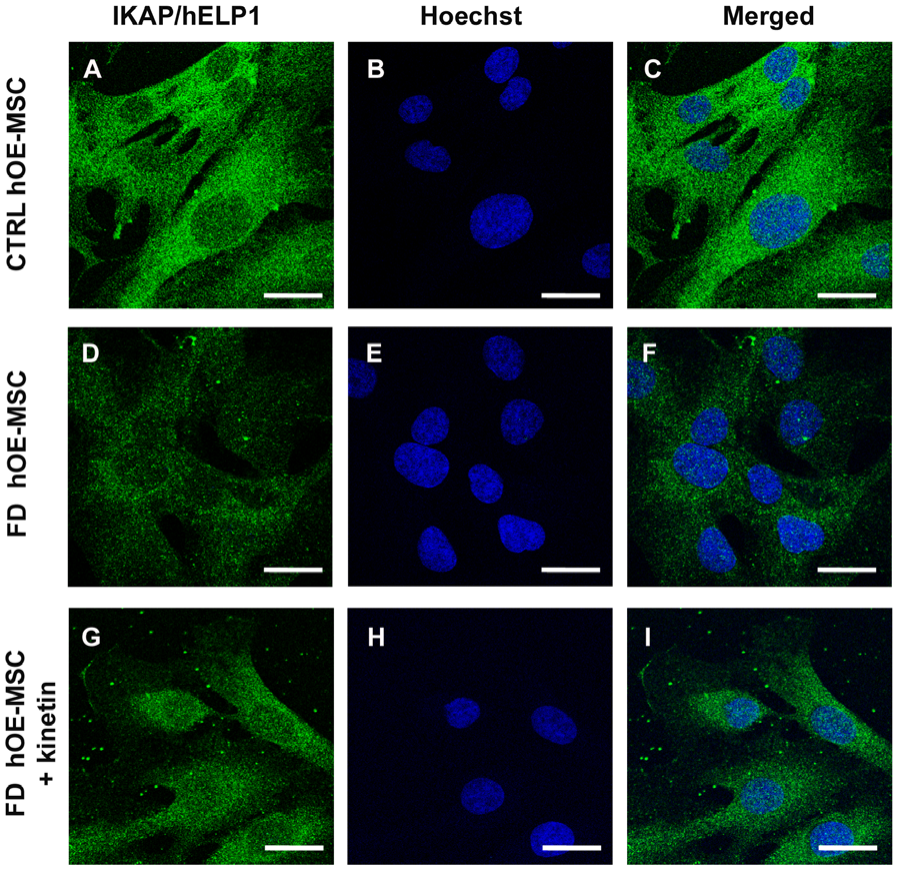
IKAP/hELP1 distribution in hOE-MSCs. Anti-IKAP/hELP1 immunofluorescence staining in control (A, B, C), FD hOE-MSCs (D, E, F), and FD hOE-MSCs treated with 100 µM kinetin for 24h (G, H, I). The primary antibody used is a mouse monoclonal anti-IKAP/hELP1. Scale bars represent 20 µm.

Since the localization of IKAP/hELP1 remains controversial and is important to understand protein functions, we stained both control and FD hOE-MSCs with the monoclonal antibody directed against IKAP/hELP1 and previously used for detecting the protein by western blot analysis. In control cells, confocal imaging revealed a weak and diffuse signal with a dominant cytoplasmic staining within the perinuclear area. We could also detect the presence of IKAP/hELP1 in the nucleus of hOE-MSCs (Figure 3A–C). Significantly, FD hOE-MSCs exhibit a weaker anti-IKAP/hELP1 immunofluorescence staining compared to control cells, with a similar distribution of the staining (Figure 3D–F). Therefore, collectively, our results are in agreement with a wide distribution of IKAP/hELP1, including a much lower IKAP/hELP1 staining in FD hOE-MSCs, in agreement with RT-qPCR and western blot analysis.

### Transcriptome analysis identified fifty dysregulated genes

It is widely accepted that culture conditions alone may exert effects on gene expression, resulting in experimental inconsistencies [38], [39]. Thus, to investigate the involvement of candidate disease mechanisms in FD and to test whether differences in gene expression are stably imprinted in FD compared to control hOE-MSCs, we explored the transcriptome of these cells at very early (P1, P2) and later (P5, P9) cell passages with the same samples used to quantify *IKBKAP* transcripts. Among the 8,780 cDNA represented on the microarray, 46 were significantly decreased and only 4 increased in FD hOE-MSCs, when compared to control hOE-MSCs (fold-change>1.4-fold; p-value<6.10^3^, Table 1 and Table S1), considering a false discovery rate (FDR) of 3% (Figure S1). Notably, the biological processes and the signaling pathways most significantly targeted by the effectors on our list were actin cytoskeleton organization, cell growth, and apoptosis (Table 1). More specifically, we identified 10 genes (Table 1 and Table S1) that also exhibited a significant dysregulated expression in previous microarray studies [10], [22]. Interestingly, 2 genes, *PMEPA1* and *GSN* (encoding TMEPAI and gelsolin, respectively), involved in cell growth and cytoskeleton organization, respectively, were dysregulated in both the *IKBKAP* RNAi and FD iPS cell studies.

**Table 1:** Most dysregulated genes in FD hOE-MSCs are involved in seven over-represented cellular processes.

Gene	Clone ID	FC	*p*-value	Biological process	Studies
				**Actin cytoskeleton reorganization ** ***p*** ** = 0.000275**	
*GSN*	246170	−1.91	0.00124	actin cytoskeleton reorganization	*1 *2
*MYO9B*	279085	−1.70	0.00144	actin cytoskeleton remodeling	
*MYPN*	325601	−1.52	0.00131	sarcomere organization through nebulin and α-actinin interactions	
*DSTN*	149199	−1.48	0.00017	actin filament depolymerization	
*CORO2B*	547561	−1.44	0.00079	neuronal actin structure reorganization	
*SLC9A3R2*	155467	2.42	0.00034	adaptor of ion channels and receptors to the actin cytoskeleton	*1
				**Regulation of apoptosis ** ***p*** ** = 0.00203**	
*CHEK2*	1893020	−2.20	0.00007	cell cycle arrest and apoptosis in response to DNA damage	*1
*ZMAT3*	525407	−1.78	0.00251	positive regulation of p-53-mediated apoptosis	
*TNFSF10*	713945	−1.75	0.00041	induction of apoptosis by activation of caspase activity	*2
*PARP3*	436086	−1.60	0.00004	positive regulation of apoptosis- maintenance of genomic stability	
				**Transport ** ***p*** ** = 0.00224**	
*ABCG5*	121977	−1.59	0.00089	cholesterol transport in and out of the enterocytes	
*SLC35E1*	487960	−1.55	0.00086	monosaccharide transport	
*SLC22A6*	36482	−1.52	0.00050	α-ketoglutarate transmembrane transporter activity	
*SFXN2*	757192	−1.45	0.00021	iron transport	
				**Cell proliferation ** ***p*** ** = 0.00552**	
*APOE*	1870594	−1.68	0.00028	cell proliferation-regulation of neurite extension	*1
*CD22*	284220	−1.58	0.00032	B cell proliferation	
*CD38*	123264	−1.54	0.00074	B cell proliferation	
*GBA*	757264	−1.53	0.00012	cell proliferation-ceramide metabolic process	
*SERINC2*	149995	1.84	0.00014	cell proliferation	
				**Regulation of cell growth and cell cyle ** ***p*** ** = 0,0091**	
*PMEPA1*	366599	−4.92	0.00585	EGF receptor signaling pathway - negative regulation of cell growth	*1 *2
*STRBP*	669157	−1.75	0.00007	regulation of cell growth	
*INO80B*	323554	−1.63	0.00104	growth induction and cell cycle arrest at the G1 phase	
*S100A16*	739851	−1.55	0.00012	regulation of cell cycle progression	*1
*CDIPT*	306047	−1.49	0.00088	regulation of cell growth	
				**Nervous system process ** ***p*** ** = 0,0302**	
*LRCH1*	683580	−3.62	0.00080	long-term memory and learning - signal transduction	
*KCNT2*	38677	−2.06	0.00010	synaptic transmission mediated by K (+) channels	
*NUMBL*	1855110	−1.61	0.00118	Notch signaling pathway inhibition - cerebral cortex morphogenesis	
*DULLARD*	346368	−1.53	0.00198	Nuclear organization-negative regulation of BMP signaling	
				**Proteolysis ** ***p*** ** = 0,0334**	
*FBXL15*	166240	−1.71	0.00130	ubiquitin-dependent protein catabolic process	
*WSB1*	298983	−1.61	0.00123	ubiquitination and proteosomal degradation of target proteins	
*PCSK7*	241130	−1.53	0.00155	proteolysis- ubiquitous endoprotease activity	
*RNF115*	471834	−1.42	0.00067	proteolysis- vesicle-mediated transport - vesicle traffic	
*MMP27*	767086	−1.35	0.00067	proteolysis of fibronectin, laminin, gelatins and/or collagens	

Clone ID represents the number assigned to the original clones produced by the I.M.A.G.E Consortium. FC = Fold change, and *p*-values were calculated by SAM analysis as described in Methods. This list of genes was annotated with the Explain™ System from Biobase. 7 majors processes are overrepresented in our list of genes, and, for each process, *p*-values were calculated and adjusted by the Bonferroni correction. The last column indicates the genes that were also found to be significantly dysregulated in 2 previous FD studies.

*1 = Lee et al. 2009.

*2 = Close et al. 2006.

**Figure 4:**
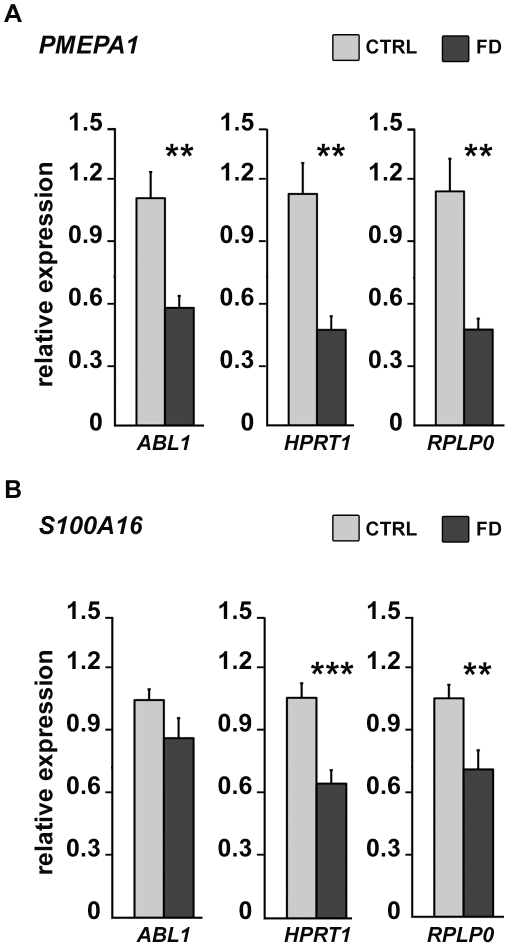
Relative levels of expression of *PMEPA1* and *S100A16* transcripts determined by RT-qPCR. RT-qPCR using total RNAs extracted from 4 controls and 4 FD hOE-MSCs at cell passages 2, 4 and 7. Histograms represent the mean value of *PMEPA1* (A) and *S100A16* (B) transcript expression level, relative to 3 reference genes *ABL1*, *HPRT1*, *and RPLP0* in control (grey) and FD samples (black). Error bars denote standard errors. (* *P*<0.05; ** *P*<0,01, *** *P*<0,001 using two-tailed Student's test).

In order to assess the robustness of our microarray analysis, RT-qPCR analysis was performed, on independent RNAs extracted from 4 control and 4 FD hOE-MSCs harvested at the second, fourth, and seventh cell passage. Since gene expression quantification using RT-qPCR requires a steady reference gene, we selected three genes frequently used for normalization of the data, *ABL1*, *RPLP0*, and *HPRT1*. We confirmed that *PMEPA1* (Figure 4A), the most dysregulated gene on the microarray, and *S100A16* (Figure 4B), were significantly underexpressed in FD samples. The expression pattern of these two candidate genes was essentially identical at all passages with the 3 reference genes, which demonstrates the validity and reliability of the array data.

### FD OE-MSCs migration is altered compared to controls

**Figure 5:**
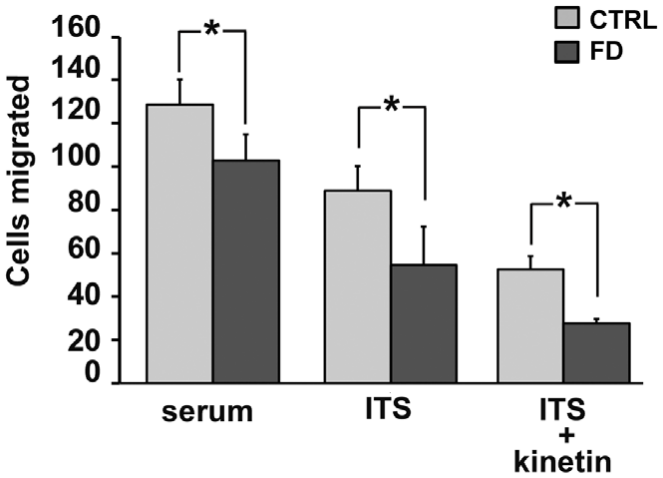
FD hOE-MSCs demonstrate reduced migration. Cell invasion in 3 different controls and 3 different FD hOE-MSCs was studied using Boyden chamber assay. Cells (3×10^4^) were added to the upper chamber in serum medium, serum-free medium (ITS), or ITS supplemented with 100 µM kinetin. Cell invasion was mesured after 24 h. Results are shown as the average ± SEM of the number of cells per microscopic field. (* *P*<0.05).

To explore the functional consequence of a down-regulated expression of genes involved in cell migration in FD hOE-MSCs compared to control cells, we used the Boyden's chamber assay. After comparing the migration pattern of 3 control and 3 FD hOE-MSCs in serum medium and serum-free medium (ITS), we determined that FD cells invasion is significantly reduced compared to control cells both in serum and in ITS medium (Figure 5).

### Confirmed down-expression of first and final *IKBKAP* exons in FD hOE-MSCs

**Figure 6:**
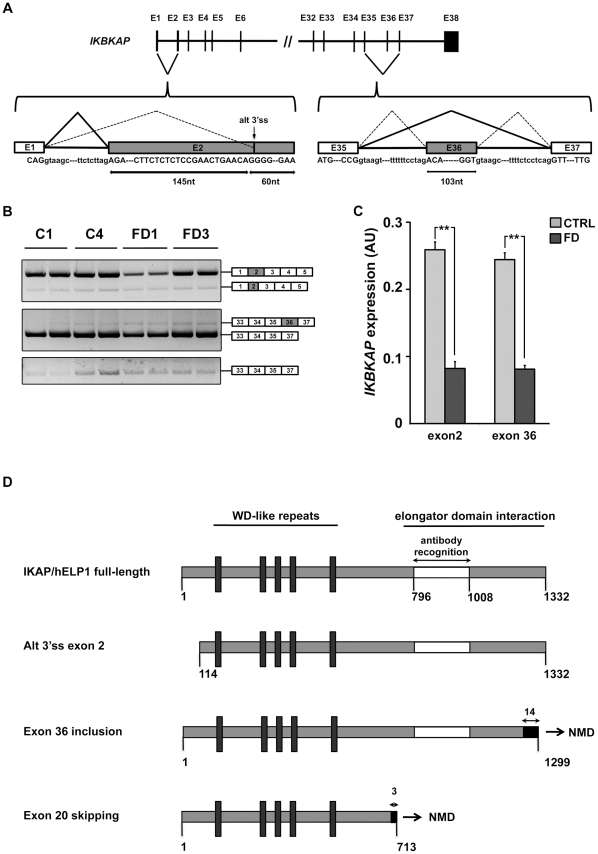
Expression and alternative splicing of *IKBKAP* mRNA at the extremities of the coding sequence. Two additional splicing events are described within *IKBKAP* gene. The first one represents the alternative use of a 3′ss for exon 2 and the second one concerns exon 36 skipping, as represented by a schematic (A). B, semi-quantitative RT-PCR illustrates relative amounts of both events on 2 control and 2 FD hOE-MSCs. C, RT-qPCR analysis performed at 2 different regions (exon 2 and exon 36), using 4 control and 4 FD samples, at the same cell passage (P7). Histograms represent the mean value of 4 samples, normalized with *ABL1* gene. (*** *P*<0,001 using two-tailed Student's test). IKAP/hELP1 truncated regions for all splicing events are represented by a schematic (D). Grey portions represent the conserved amino acids while black portions represent new amino acids resulting from a frame shift. Putative functional domains of the protein are indicated as well as the immunogenic region for the monoclonal antibody used in western blot and immunocytochemistry experiments.

Since we and others [22], [40] did not detect *IKBKAP* among the significantly down-regulated transcripts in FD compared to control samples, we asked whether this discrepancy could be due to a lack of sensitivity of microarray compared to RT-qPCR. For this purpose, we decided to analyze *IKBKAP* levels of expression by investigating other exons distal from *IKBKAP* exon 20. By looking at the beginning of *IKBKAP* transcript, we identified a second event of alternative splicing. After amplifying transcripts from exon 1 to exon 5, we obtained 2 PCR products (Figure 6B, upper panel). The sequencing of the less abundant and shorter PCR product revealed the use of an alternative 3′ss within *IKBKAP* exon 2, which is shortened of 145 nt (Figure 6A, left schematic). Accordingly, the loss of the ATG start codon located within the 5′ end of exon 2 can potentially induce the use of an alternative ATG start codon (in exon 4), resulting in the synthesis of a putative 114 amino acid-truncated IKAP/hELP1 protein (Figure 6D).

When investigating expression at the end of *IKBKAP* coding sequence, again we observed a third alternative splicing event. The amplification from exon 33 to exon previously numbered exon 36 (and now called exon 37) revealed 2 products (Figure 6B, middle panel). The sequencing of the barely detectable and longer PCR product revealed the inclusion of an additional exon (Figure 6A, right schematic). This exon inclusion also induced a frameshift and resulted in a premature stop codon whose relative location may lead to NMD of this new isoform (Figure 6D). We confirmed this exon 36 inclusion with specific primers (Figure 6B, lower panel).

Both new alternative splicing events we described were also observed in others cell types (fibroblasts, HeLa, peripheral mononuclear cells, data not shown) and we decided to focus on the two major splicing events, full exon 2 inclusion and exon 36 skipping. We derived the tools (plasmids, primers, probes) to perform absolute quantification of full-length exon 2 inclusion and exon 36 skipping by RT-qPCR on samples from 4 controls and 4 FD hOE-MSC cultures. Similar underexpression of *IKBKAP* transcripts (WT+MU) was observed in FD cells compared to control cells, regardless of the exon investigated (Figure 6C). Thus, these results confirmed a decreased level of *IKBKAP* transcripts (WT+MU) in FD cells.

In addition, we tested for the stability of exon 36-containing transcripts after cycloheximide treatment. Absolute RT-qPCR analysis revealed that the exon 36 skipping ∶ exon 36 inclusion ratio decreases when NMD pathway is inhibited (Figure S2), suggesting that transcripts including exon 36 are degraded through NMD in FD, as well as in control OE-MSCs (data not shown).

### Kinetin treatment corrects aberrant *IKBKAP* pre-mRNA splicing

**Figure 7:**
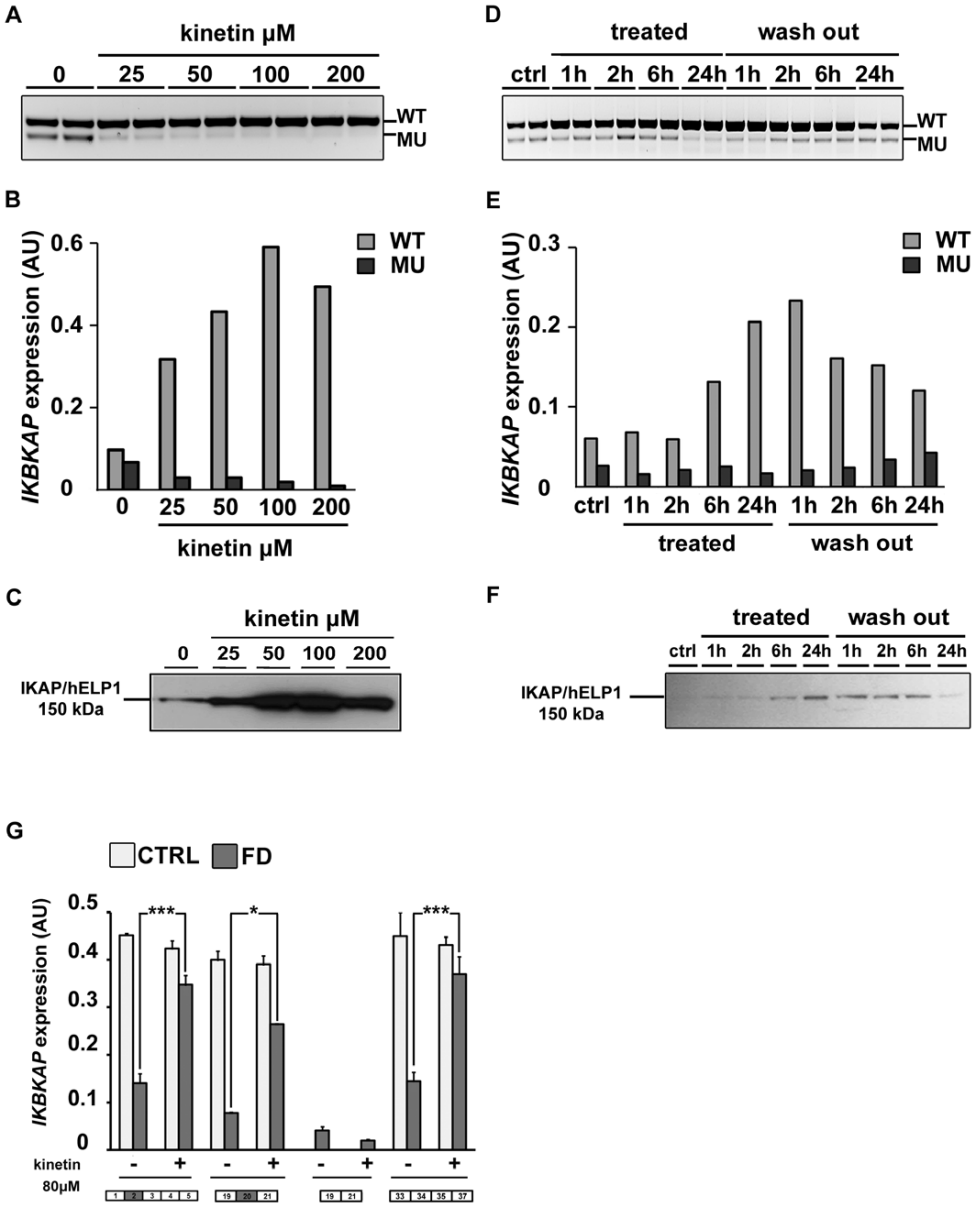
Action of kinetin on *IKBKAP* mRNA splicing in FD hOE-MSCs cells. A–C, hOE-MSCs were treated with increasing concentration of kinetin for 72 h. (i) Total RNAs were reverse transcribed and subjected to both semi-quantitative PCR (A) and absolute qPCR (B) of WT and MU *IKBKAP* transcripts, (ii) total lysates were analyzed by western blot using a monoclonal mouse anti-IKAP/hELP1 antibody (C). D–F, kinetics of hOE-MSCs incubated for 24 h with 80 µM kinetin which was then removed for the next 24 h. Total RNAs were reverse-transcribed and subjected to *IKBKAP*-specific semi-quantitative PCR (D) and absolute RT-qPCR (E). Total lysates were analyzed by western blot (F). The level of WT and MU transcripts was normalized using *ABL1* as a reference gene (B and E). G, Two controls (CTRL) and two FD hOE-MSCs treated or not with 80 µM kinetin for 24h were analyzed by absolute RT-qPCR to determine the amount of *IKBKAP* exon 2 inclusion, exon 20 inclusion, exon 20 skipping, and exon 36 exclusion after normalization with *ABL1*. (* *P*<0.05; *** *P*<0,001 using two-tailed Student's test).

Our next goal was to assess whether the production of both WT and MU *IKBKAP* mRNAs can be modulated in our model. In previous studies, one compound, kinetin (6-furfurylaminopurine) was found to correct *IKBKAP* splicing and increase IKAP/hELP1 production in FD cells [35]. We tested whether this drug could also modify the splicing defect of *IKBKAP* in FD hOE-MSC cells. For this purpose, we used increasing concentrations of kinetin (25 to 200 µM) on a FD hOE-MSCs culture for 72 h. As expected, after semi-quantitative RT-PCR, we observed a significant decrease of MU transcript compared to non-treated cells on agarose gel electrophoresis (Figure 7A). The level of *IKBKAP* mRNA splicing correction increased proportionally to the concentration of kinetin, and the MU transcript almost vanished at 100 µM. The dose-dependent action of kinetin on increasing WT∶MU ratio was confirmed by RT-qPCR analysis (Figure 7B). A similar finding was observed when IKAP/hELP1 proteins were detected by western blot analysis (Figure 7C). Accordingly, when FD hOE-MSC were incubated with 100 µM kinetin for 24 h, we observed a major increase of anti-IKAP/hELP1 staining, in cytoplasmic as well as in nuclear areas (Figure 3G–I). However, the same kinetin treatment could not rescue the migration defect observed in FD hOE-MSCs with the Boyden's chamber assay (Figure 5).

In order to determine how fast kinetin modulates *IKBKAP* mRNA splicing, we performed a time-course experiment with a constant concentration of 80 µM over 24 h. After performing semi-quantitative RT-PCR analysis, the first significant increase of WT∶MU ratio was seen after 24 h of kinetin treatment (Figure 7D). However, quantitative analysis by RT-qPCR revealed that kinetin significantly enhances the ratio 6 h after its addition to the culture (Figure 7E). Interestingly, time-response of kinetin was maximal at 24 h during treatment, but its effect on splicing lasted more than 6 h after the drug was washed out and WT transcript levels remained high compared to non-treated cells, at least 24 h after the wash out. Consistent results were observed for IKAP/hELP1 protein expression by western blot analysis, although a strong decrease of protein amount appeared after 24 h of wash out (Figure 7F).

Finally, we wanted to investigate kinetin activity along the *IKBKAP* transcript. Therefore, we compared the level of expression of *IKBKAP* transcripts by RT-qPCR to focus on different transcript regions for both control and FD hOE-MSCs, with or without the presence of 80 µM kinetin for 24h. We first observed that the total amount of *IKBKAP* transcript detected was almost identical when probes at the extremities or in the middle of the transcript were used (Figure 7G). In addition, kinetin has no significant effects on *IKBKAP* transcript levels in control cells, which likely excludes a potential action of kinetin on *IKBKAP* transcription. Moreover, kinetin, by improving *IKBKAP* exon 20 recognition, restores *IKBKAP* transcript levels in FD hOE-MSCs similar to those observed in control cells (Figure 7G). Kinetin did not modify the ratio of alternative splicing around exon 2 and exon 36, suggesting its specific mechanism of action on exon 20 inclusion (data not shown).

Altogether, these results revealed that kinetin exerts a rapid and possibly long lasting effect on *IKBKAP* mRNA splicing, which most likely occurs by increasing *IKBKAP* mRNA stability rather than acting on transcription.

### FD sphere cells display a strongly reduced *IKBKAP* exon 20 skipping

One property of multipotent cell consists in their capacity to organize into spheres when cultured in appropriate medium. Since FD hOE-MSCs express a significant amount of MU *IKBKAP* transcript we asked whether induction of sphere formation could modify the WT∶MU *IKBKAP* transcripts ratio.

**Figure 8:**
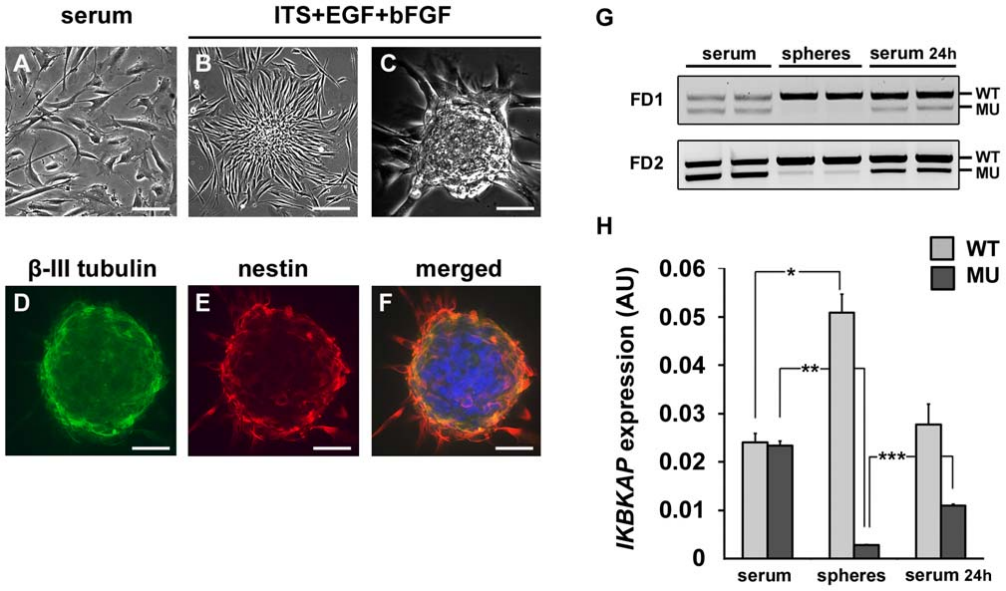
WT∶MU ratio is increased in hOE-MSC-derived spheres. FD hOE-MSCs cultured in serum (A) gave rise to spheres when plated in medium supplemented with ITS and growth factors (B and C). Immunostaining showed β-III tubulin and nestin positive spheres (D, E, F). RNA was isolated from 2 different FD hOE-MSCs cultured first in serum, then induced to form spheres, and finally dissociated and replated in serum conditions for 24h (G). RT-qPCR was performed on the same samples and histograms represent the mean value of the two FD samples after normalization with *ABL1* gene (H). Scale bars represent 100 µm. (* *P*<0,05, ** *P*<0,01 using two-tailed Student's test).

Although hOE-MSCs proliferate as adherent cells, when cultured in DMEM-F12 supplemented with serum (Figure 8A), they progressively organize into spherical aggregates when cultured in serum-free medium in the presence of EGF and bFGF (Figure 8B and C). Both control and FD hOE-MSCs were able to form spheres in approximately one week, and immunostaining with anti-β-III tubulin (Figure 8D) and anti-nestin (Figure 8E) antibodies revealed a similar staining of both markers for control and FD cells (Figure 8F). Total RNAs, isolated from either FD spheres, FD cells cultured in serum during the same period, or dissociated cells from spheres that were reintroduced in serum medium for 24 h, were subjected to RT-qPCR. We observed a significant increase of *IKBKAP* exon 20 inclusion in spheres, when compared to hOE-MSCs in serum conditions, as well as a semi-disappearance of *IKBKAP* exon 20 skipping (Figure 8G). Dissociated spheres re-exposed to serum rapidly expressed initial levels of WT and MU transcripts (Figure 8G). We quantified WT and MU transcript level of expression in these 3 different conditions and confirmed that spheres formation from FD hOE-MSC induces *IKBKAP* mRNA splicing correction, using RT-qPCR (Figure 8H). We also looked for exon 2 and exon 36 alternative splicing events but did not detect significant alterations of splicing ratio resulting from sphere formation and dissociation (data not shown).

### Commitment of FD OE-MSCs into neuronal and glial lineages leads to a more severe *IKBKAP* exon 20 skipping

**Figure 9:**
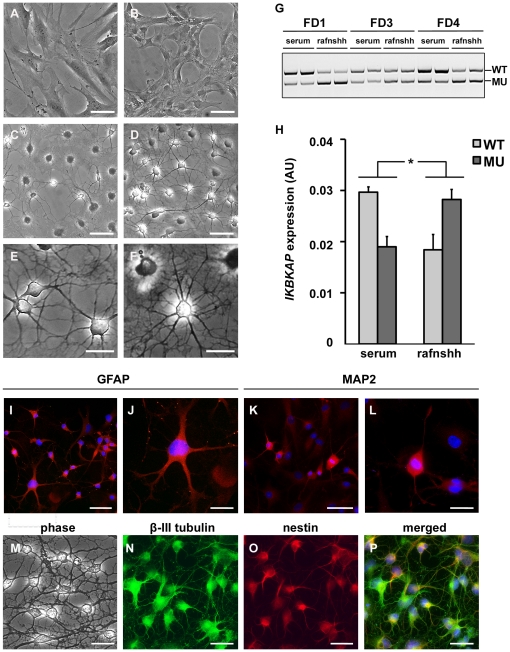
WT∶MU ratio is decreased in differentiated hOE-MSCs. A, Phase contrast microscopy of FD hOE-MSCs cultured for 48 h in either serum (A), or N2B27 conditions (B), or with the rafnshh cocktail including retinoic acid, forskolin and Sonic hedgehog (C), or rafnshh for 7 days (D). Details of connections established between cells and extensive cellular arborization after 7 days in rafnshh condition are shown in E and F. G, agarose gel electrophoresis of semi-quantitative RT-PCR products showing *IKBKAP* WT and MU transcripts of 3 differents FD patients before and after rafnshh treatment. H, histograms represent the mean level of *IKBKAP* transcripts expression normalized with *ABL1* gene expression for 3 FD patients, after RT-qPCR analysis. (* *P*<0,05, using two-tailed Student's test). FD hOE-MSCs treated for 7 days in rafnshh were fixed and stained for GFAP (I, one cell with ramified neuritic process is magnified in J), MAP2 expression (K and L). M–P, double labelling of rafnshh-treated cells with anti-β III tubulin and anti-nestin antibodies. Scale bars represent either 50 µm (A–D, I, K, M–P) or 25 µm (E–F, J, L).

FD hOE-MSCs were treated for 7 days to induce neuronal differentiation with a protocol previously used in hOE-MSC [41], which consists of additing retinoic acid, forskolin, and Sonic hedgehog in the medium (called rafnshh medium). Cells were first cultured in serum-free medium supplemented with N2 and B27 until they became adherent before being cultured in rafnshh (Figure 9B). The new culture medium induced a slight morphology change, as compared to the serum condition (Figure 9A). When hOE-MSCs were first cultured in rafnshh, they began to form long fine processes and neural-like cells (Figure 9C). After 7 days of treatment, a majority of cells adopted neuron-like morphologies (Figure 9D) and established a wide range of connections (Figure 9E, F and M). Using end-point PCR on 3 different FD cell cultures, we observed that *IKBKAP* mRNA splicing in rafnshh-treated cells was more prone to exon 20 skipping as compared to untreated cells (Figure 9G). This change can be quantified by RT-qPCR (Figure 9H). In contrast, we did not detect significant variations in exon 2 and exon 36 alternative splicing during neuronal differentiation (data not shown).

When assessing immunostaining on treated cells, we observed that rafnshh treatment increased the proportion of both GFAP (Figure 9I and J) and MAP2-expressing cells (Figure 9K and L). Double-labeling with β-III tubulin and nestin revealed a stronger expression of β-III tubulin compared to nestin during the differentiation process (Figure 9N–P). Collectively, these results indicate that rafnshh treatment influences the neural and glial lineage commitment. As a consequence, the splicing machinery in neuron or astrocyte-differentiated cells is impaired for *IKBKAP* exon 20 recognition.

## Discussion

Deciphering the molecular basis of the tissue-specific pattern of *IKBKAP* mRNA splicing in FD nervous tissues is crucial for the comprehension of disease physiopathology in this genetic neurological disorder affecting neuronal development and survival. In this study, we aimed to recapitulate different aspects of *IKBKAP* gene expression using FD hOE-MSCs cultured with different conditions. While other human cellular models, such as fibroblasts or iPS cells, have been investigated to understand FD, we believe that hOE-MSCs hold a great promise to model the FD disease pathology. hOE-MSCs are easily obtained by a simple biopsy and can be maintained for an extended period of time and can be rapidly expanded in basic culture conditions without genetic manipulation. In addition, due to the origins of hOE-MSCs from a peripheral tissue, these cells are able to express neuroglial markers *in vitro* (Figure 1, [42]–[47]). Thus, they constitute an efficient and simple method to derive neuronal cells in the original context of the genetic mutation studied.

In contrast to control cells, which constitutively include *IKBKAP* exon 20, we confirmed the alternative splicing of that exon in FD cells (Figure 2A). Similar to neural precursors obtained from iPS cells [22], FD hOE-MSCs predominantly express the MU *IKBKAP* transcript isoform (Figure 2A and B). Moreover, we demonstrated that FD cells exhibit notably lower *IKBKAP* transcript levels (WT+MU), when compared to controls (Figures 2B, 6C and 7G). Such a difference is most likely explained by an extensive degradation of MU transcripts through the NMD pathway (Figure 2D), as was previously suggested [35]. However, NMD efficiency varies between cell types and individuals [48]–[50] and it is unclear how prevalent this mechanism of mRNA degradation is in the nervous system of FD individuals. In order to get a better insight into the actual contribution of NMD on the decay of *IKBKAP* MU transcripts, it will be necessary to specifically block the NMD machinery.

So far, the proposed functions of IKAP/hELP1 are related to various cellular localizations. This has been the matter of a controversy because several studies have failed to detect IKAP/hELP1 in the nucleus or found it almost exclusively in the cytoplasm [11], [21], [34], [51], which is difficult to reconcile with its suggested role in transcription elongation. As observed in most published studies, we observed that the immunolocalization of IKAP/hELP1 was mainly cytoplasmic within the perinuclear area. However, we also detected significant nuclear staining in agreement with other reported studies [9], [52]. Altogether, our findings of IKAP/hELP1 distribution in hOE-MSCs support multiple roles for the protein within different subcellular compartments.

In order to establish a direct link between low levels of *IKBKAP* WT transcripts and decreased neuronal populations in FD patients, several groups have investigated transcriptome variations resulting from a reduced level of *IKBKAP* transcripts using microarray technology [10], [22], [34], [40], [53]. However, poor correlations were observed between these studies. Several reasons can explain these discrepancies. First, various cell types, used at different stages of development and differentiation, have been studied (brain tissue, fibroblasts, HeLa cells, HCT116 cells, iPS cells). The cells tested in the current study are likely at a stage between the iPS and iPS cell-derived neural crest precursors developed by the Studer group [22]. It is thus not surprising that most of our microarray results overlap with that of the iPS cell study. Second, a potential source of variability among transcriptome analyses derives from the technical manipulations employed to downregulate *IKBKAP* (presence of the FD mutation in its original context compared to WT *IKBKAP* knockdown using different interfering RNAs), resulting in differential residual *IKBKAP*/IKAP expression. Third, in the context of a rare disease, a small sample size may cast doubt upon the validity of drawn conclusions. To decrease statistical bias, we decided to increase the number of samples of our 5 control and 4 FD patients by collecting data from 4 different passages (P1, P2, P5 and P9) of each primary cell line. We hypothesized was that such a method would allow us to i) increase the statistical power of our analysis, and ii) explore the effects of culture conditions on gene expression. We anticipated that a strong gene dysregulation observed in microarray would be more significant if this expression is stably maintained at any cell passage. Most of the differentially expressed genes were found to have a modest (<2) fold-change (Table 1). Interestingly, like previous studies, we observed that a majority of genes were down-regulated in FD hOE-MSCs (Table 1, negative values), and only 4 genes were up-regulated (Table 1 and Table S1, positive values). This observation is in agreement with other studies [10], [22], [40] and may reflect a defect in transcription due to decreased Elongator activity, as previously proposed [10]. Importantly, 10 genes in our list (20%) appeared to be correlated with one or two previous investigations (Table 1 and Table S1, last column). In one of the past studies, *IKBKAP* expression level can be downregulated by RNAi in control cells [10], where there is no production of MU transcripts. Thus, different studies share dysregulated genes in different contexts of either constitutive or alternative splicing of *IKBKAP* mRNA. This suggests that *IKBKAP* alternative splicing may not be the only pathological alteration in FD.

Similar to what was previously reported, our study revealed the downregulation of gelsolin (*GSN*), a protein involved in cell motility, that causes defects in cytoskeleton reorganization and cell migration in FD [10], [11], [22], [34]. The most dysregulated gene observed in our study was *PMEPA1* (−4.92 fold change), encoding the TMEPAI protein which has recently been reported to be a direct target of the TGF-β signaling pathway and is involved in cell growth, cell differentiation, and apoptosis [54]. Due to its important cellular function and repeated reports of its dysregulation in FD cells, it would be very interesting to test TMEPAI in further studies. In agreement with previous studies correlating a decreased expression of IKAP/hELP1 with defects in cell migration [10], [11], [22], [34], the Boyden's chamber assay show that FD hOE-MSCs have decreased migration potential, compared to control cells (Figure 5).

Surprisingly, we did not find *IKBKAP* as a dysregulated gene in our microarray analysis. This result is all the more intriguing since this gene is expressed at much lower levels in FD hOE-MSCs, as shown by RT-qPCR in the exon 20 region (Figure 2B). However, previous analyses using microarrays also failed to detect *IKBKAP* as a down-regulated gene in FD cells [22], [40]. To address the question of a possible PCR artifact or lowered microarray sensitivity and because the FD mutation is located in the middle region of the *IKBKAP* gene, we performed quantitative PCR at both ends of the *IKBKAP* gene.

Unexpectedly, we identified 2 new events of alternative splicing at both extremities of *IKBKAP* coding sequence (Figure 6A). However, these results are in agreement with EST sequences found in alternative splicing database such as ASD [55]. We revealed that the alternative use of a 3′ss (downstream of the ATG start codon) leads to a shorter exon 2 which can potentially induce the use of an alternative ATG start codon (in exon 4), resulting in the synthesis of an N-terminal truncated IKAP/hELP1 protein. In addition, we detected the presence of intronic sequences at the end of *IKBKAP* gene leading to a supplementary exon in the mRNA, named “exon 36”. This exon inclusion also induced a frameshift and resulted in a premature stop codon whose relative location likely led to NMD of this new isoform, as observed by stabilization of the transcript after cycloheximide treatment (Figure S2).

IKAP/hELP1 plays the role of a scaffold protein in Elongator complex assembly and the C-terminus half of IKAP/hELP1 is responsible for this function [34]. It has also been shown that IKAP/hELP1 contains five WD-like repeats domains in the N-terminal part that may play a role for protein-protein interactions [56]. When comparing the different protein isoforms resulting from the 3 alternative splicing events we described (Figure 6D), only the isoform resulting from exon 20 skipping seems to lack a functional domain and may play a pathological role during FD progression. However, the protein domains of IKAP/hELP1 important for Elongator integrity have not been precisely mapped and other roles for IKAP/hELP1 have been proposed outside of the Elongator complex. Thus, it is not clear whether the new *IKBKAP* isoforms we described, may have functional roles. Future investigations with specific reagents (antibodies) will be required to address this issue.

Nevertheless, we consistently detected a lower expression of *IKBKAP* gene (including the full length exon 2 transcript and the transcripts skipping exon 36) in FD hOE-MSCs (Figure 6C), as determined when investigating the exon 20 region. Thus, the relatively stable expression of *IKBKAP* observed in microarray analysis may be due to a weak expression that is masked within the noise signals. Furthermore, during the analysis of our microarray data and those of previous studies [10], [22], [40] we noticed that a high fraction of genes were expressed at background levels. This points to the limitation of using microarray technology to establish the whole genome expression pattern. We expect that new technologies such as RNA deep sequencing will rival PCR sensitivity and specificy in the near future.

The model of hOE-MSCs from FD patients has also been very useful to test compounds, such as kinetin, that can correct the defective splicing process. As reported in the other cell types tested, we confirmed that kinetin corrects splicing in a dose-dependent manner in FD hOE-MSCs (Figure 7A–C). This suggests that kinetin activity is not cell-type specific. Although the mechanism by which kinetin modulate splicing remains poorly understood [36], [57], it is unlikely that kinetin acts directly on the general transcription machinery as the level of *IKBKAP* transcripts was not significantly modulated by kinetin in control hOE-MSCs (Figure 7G). This effect of kinetin has also been previously observed in control iPS cells [22]. Time-course experiments of kinetin treatment revealed that the drug acts quite rapidly on correcting *IKBKAP* mRNA splicing and enhancing IKAP/hELP1 synthesis, but its effects last only a short time after removal (Figure 7E and F). This information provides new perspectives in the strategy of kinetin delivery to FD patients. First, kinetin as an FD treatment would potentially decrease deleterious consequences of the mutation at the protein level. In addition, drug efficacy may be achieved if adequate levels of kinetin is maintained over a long period of time. However, as observed for FD iPS cells [22], kinetin did not improve cell migration in FD hOE-MSCs (Figure 5), suggesting incomplete phenotype complementation.

Using the hOE-MSCs model, we were also able to modulate the expression of *IKBKAP* WT and MU transcripts, by exposing the cells to different culture conditions to simulate variations in alternative splicing occurring during development and differentiation. hOE-MSCs form free floating spheres in approximately 7 days, when cultured with EGF and bFGF in serum-free medium. It is known that in sphere conditions, cells can form a niche prevent differentiation and ensure self-renewal. The cell populations contained in hOE-MSC-derived spheres are not well-known. Some reports indicate that they include an heterogeneous mixture of stem cells and neuroglial progenitors [29], [43], [44], [47], [58]. However, immunostainings of nestin and β-III tubulin show no significant differences. Interestingly, PCR analysis demonstrated that spheres express higher levels of WT *IKBKAP* transcript compared to hOE-MSCs in serum and express very flow levels of MU transcript. However, when the cells were transferred back to culture conditions with serum, the enhanced *IKBKAP* exon 20 inclusion was not maintained (Figure 8G and H). FD hOE-MSCs that were cultured in serum-free conditions without forming spheres did not exhibit significant changes in *IKBKAP* isoforms, suggesting that there is subpopulation of cells within the spheres that can promote *IKBKAP* exon 20 inclusion. These results indicate that when FD cells are turned back into a more “primitive” developmental stage, *IKBKAP* aberrant splicing is corrected, as was described during the fibroblast to iPS cell reprogramming process [22]. Accordingly, commitment into a more differentiated neuronal state would alter *IKBKAP* exon 20 inclusion. Therefore, we differentiated FD hOE-MSCs, using a previously established protocol which included retinoic acid (RA), forskolin (FN), and Sonic hedgehog (Shh) in the culture medium [44]. In these conditions, we observed that differentiated cells express the highest levels of MU *IKBKAP* transcript (Figure 9G and H). This result correlates with the specific low WT∶MU *IKBKAP* isoform ratio in nervous tissues [8], and suggests that stem cells engaged in a neuronal lineage with appropriate culture conditions can rapidly switch their *IKBKAP* WT∶MU transcript ratio.

Previous studies have shown that i) *IKBKAP* exon 20 is poorly defined in a “healthy” context, due to the presence of a weak 3′ss and exonic splicing silencers, and ii) the FD mutation exacerbates the environment leading to alternative exon 20 inclusion in FD tissues [59], [60]. We propose that some transcription/splicing factors involved in *IKBKAP* exon 20 recognition are also downregulated in a tissue-specific manner. This would explain why the pattern of *IKBKAP* alternative mRNA splicing is more aberrant in the nervous system. Interestingly, Lee and colleagues determined that the neuron-specific splicing factor NOVA1 [61] was underexpressed in FD versus control iPS cell-derived neural crest precursors [22]. The new model described in this study will allow us further test whether candidate splicing factors may be involved in the tissue-specific regulation of *IKBKAP* mRNA alternative splicing.

## Materials and Methods

### Ethics Statement

All control and FD participants gave informed and written consents (provided by the parents for the children) and biopsies were obtained under a protocol, which was approved by the local ethical committees in New York (Institutional Review Board of the New York University School of Medicine) and Marseille (Comité Consultatif de Protection des Personnes dans la Recherche Biomédicale Marseille 2).

### Purification of hOE-MSCs

Human nasal mucosae were obtained from biopsies of 4 FD patients (3 females and 1 male aged 12–16 years) at the Dysautonomia Treatment and Evaluation Center, New York. All four FD patients were homozygous for the splicing mutation. Biopsies form 5 healthy controls (3 females and 2 males, aged 18–39 years) were collected by the ENT Department in Marseille (Hopital Nord, France). Biopsies were harvested as previously described [30], [46] to obtain a cell culture of hOE-MSCs. The cells were continuously cultured in DMEM/HAM'S F12 (Gibco) supplemented with 10% fetal bovine serum (FBS) and 50 µg/ml gentamicin (Gibco) and trypsinized once a week with 0.05% trypsin-EDTA (Gibco) at 60–80% confluence. Cycloheximide (Sigma), diluted in DMSO, was used at 50 µg/ml. Kinetin solution (Sigma, 1 mg/ml) was diluted in DMEM/HAM'S F12 at concentration ranging from 25 to 200 µM for various incubation times, as specified in the text.

### Generation of spheres and cell differentiation

Cells were plated at 15,000 cells/cm^2^ into 6-well plates preteated with poly-L-lysine (5 µg/cm^2^, Sigma) in a serum-free medium of DMEM supplemented with insulin-transferrin-selenium (ITS, 1g/l insulin, 0.55 g/l transferrin, 0.67 mg/l sodium selenite; Gibco), epidermal growth factor (EGF, 50 ng/ml, R&D system) and basic fibroblast growth factor-2 (bFGF, 50 ng/ml, R&D system). Half of the medium was changed every 2 days. Multipotent spheres were obtained after 1 week and harvested by aspiration of the culture medium and centrifugation (5 min, 300g). They were then incubated in Accumax solution (Sigma), for 10 min at 37°C. To release more cells, the sample was gently triturated by repeated pipetting. When disaggregation was complete, cells were centrifugated (5 min, 300g) to remove cell debris. For cell differentiation, hOE-MSCs were plated on glass coverslips at the density of 10,000 cells/cm^2^ (in six-well plates for RNA extraction, and 24-well plates for immunostaining), in serum-free medium supplemented with 1% ITS, 1% B27 and 0.5% N2, until adhesion. Cells were then treated with 1% ITS, 1 µM *all-trans* retinoic acid (Sigma), 5 µM Forskolin (R&D Systems), 15 nM Sonic hedgehog (R&D Systems), 1% B27 and 0.5% N2 for 7 days without changing the medium.

### Immunocytochemistry

Cells grown on glass coverslips were fixed with 4% paraformaldehyde for 20 min at room temperature and rinsed three times with phosphate-buffered saline (PBS). Cells were preincubated for 60 min at room temperature with blocking buffer (3% BSA in PBS with 0.1% Triton X-100 and 10% normal goat serum), followed by incubation with the primary antibodies diluted in the blocking buffer. Coverslips were processed for immunofluorescence staining using the following primary antibodies: rabbit anti-nestin (1∶500, Abcys), mouse anti βIII-tubulin (1∶500, Sigma, clone SDL.3D10), rabbit anti-GFAP (1∶500, Dako), rabbit anti-MAP2 (1∶500, Abcam), mouse anti-IKAP/hELP1 (1∶100, BD Biosciences, clone 33). Each primary antibody was applied for 2 h at room temperature. For IKAP/hELP1 staining, primary antibody was incubated 3 h at room temperature followed by an overnight incubation at 4°C. We used appropriate secondary antibodies: goat anti-rabbit IgG conjugated with AlexaFluor 594 (1∶500, Invitrogen), goat anti-mouse IgG conjugated with AlexaFluor 488 (1∶500, Invitrogen) for 1 h at room temperature. Hoechst nuclear dye was used to label nuclei (1∶2,000, Molecular Probes, #33258). Coverslips were finally mounted with anti-fading medium (ProLong®, invitrogen). Cells were observed under a Nikon Eclipse E800 upright microscope equipped with epifluorescence and TRITC, FITC and DAPI filters, and images were analyzed using an Orca-ER CCD camera (Hamamatsu Photonics) and the LUCIA image analysis software (Laboratory Imaging). Confocal image acquisition was performed on a Leica TCS SP2 confocal microscope (Leica Microsystems) using the 488-nm band of an argon laser for excitation of Alexa 488 and the 680-nm band of an argon laser for excitation of Alexa 680. High magnification images were acquired using a 63× HCX PL APO (with 4 digital zoom factor) oil immersion objective (numerical aperture 1.32) by sequential scanning to minimize the crosstalk of fluorophores. Pinhole size was set to ‘Airy one” to achieve the best possible resolution (theoretical lateral and axial limits: 165 and 330 nm, respectively). Voxel size was set to 58 nm in x and y and to 162 nm in z.

### Western blot analysis

Cells were harvested by trypsination and centrifugation (5 min, 300g). The pellet, containing approximately 10^6^ cells, was resuspended in 0.5 ml 2× Laemmli buffer (0.5 M Tris pH 6.8, 4.4 ml Glycerol, 20% SDS, 1% Bromophenol Blue, 0.5 ml β-mercaptoethanol). 30 µl of cell lysates were separated on 6.5% SDS-polyacrylamide gel electrophoresis and transferred to a nitrocellulose membrane (Amersham Biosciences). After blocking with 5% nonfat milk in PBS, 0.1%, tween 20 (PBST) buffer, blots were probed for 1h at room temperature with a mouse monoclonal anti-IKAP antibody (1∶5,000, BD Biosciences, clone 33) in PBST, followed by incubation with horseraddish peroxidase-conjugated goat anti-mouse IgG (1∶5,000, Jackson Immunoresearch) for 45 min at room temperature. As a control, the membrane was also probed for β-actin (1∶3,000, Sigma, clone SDL.3010). Proteins were visualized by chemiluminescent detection using the ECL detection kit (Enhanced Chemiluminescence, Amersham) and films were digitized and analyzed using the Bio 1D software.

### RNA Isolation and semi-quantitative reverse transcription-polymerase chain reaction analysis

Total RNA was isolated using the RNeasy Mini Kit (Qiagen) with DNAse treatment on the column according to manufacturer's recommendation. Total RNA was subjected to reverse transcription (RT) using the High-Capacity cDNA Archive Kit (Applied Biosystems). End-point polymerase chain reaction (PCR) analysis was performed using the Go-Taq polymerase system (Promega) and *IKBKAP*-specific primers listed in Table 2. PCR products were separated on a 1.7% agarose gel by electrophoresis in 1× TBE buffer (Tris 0.89 M, boric acid 0.89 M and EDTA 0,02 M). DNA was visualized under UV light after ethidium bromide incorporation and documented using BioVision Camera.

**Table 2:** Sequence of primers used for end-point and TaqMan real-time PCR.

Primer Probe	Sequence	T (°C)^a^	Amplicon size (bp)	Splicing events
**End-point PCR**				
hIKBKAP ex17-18F	TCATCAATGACATTGAGGTTG	55	446 (WT)	ex20 incl/skip
hIKBKAP ex22R	ATGATTCACAGAATCTATCTG		372 (MU)	
hIKBKAP ex1F	CCGGACGCACCTCTGTTTG	60	485	alt 3′ss for ex2
hIKBKAP ex4-5R	TCAGGGTCTGTTGACCTGTG		340 (alt 3′ss ex2)	
hIKBKAP ex33-34F	TCCAGGATATCAGCGAGATC	59	449	ex36 incl/skip
hIKBKAP ex37R	GCTGATAAGATGCCATGATAC		346 (- ex36)	
hIKBKAP ex35-36R	TTGGGACCTAGAACACCTGT	59	414	ex36 incl
**Real-time PCR**				
hELP1 ex19F	GGTTCACGGATTGTCACTGTT	60	133	ex20 incl
hELP1 ex20-21R	ACATAAGTTTGTCCAACCACTTCC			
P-WTELP1 ex20R	AAACCAGGGCTCGATGATGAACA			
hELP1 ex19-21F	GGACACAAAGCTTGTATTACAGACTTA	60	121	ex20 skip
hELP1 ex21-22R	CCACATTTCCAAGAAACACCT			
P-MUELP1 ex21F	AGAGGCATTTGAATGCATGAGAAAGC			
hELP1 ex2F	CCAGGGAATCCTCAGTGCT	60	104	full length ex2 incl
hELP1 ex2-3R	TTCACTTCTCTTGAGACAGGGTCTAC			
P-WTELP1 ex2F	TCCGACTGAACAGGGGACGGT			
hELP1 ex35-37F	CAGCTACCCCGGTTCTAGGT	60	128	ex36 skip
hELP1 ex38R	GGTTCTTCTGTTGATCTTTGGTG			
P-WTELP1 ex37-38R	AAGCTCAGCATCAAGAACAGGAACC			

a Annealing temperature.

### Plasmid calibrators

A fragment of WT *IKBKAP* cDNA, containing exon 19-exon 20-exon 21 and the 16 first nt of intron 21, was cloned into pcDNA 3.1 TOPO vector (Invitrogen) and named *IKBKAP* cDNA cal. Similarly, a piece of MU *IKBKAP* cDNA, containing exon 19-exon 21 and the 19 first nt of exon 22, was cloned into a pcDNA 3.1 TOPO vector and named *IKBKAP* skipEx20cal. A piece of WT *IKBKAP* cDNA, containing the last 103 nt of exon 35-exon37-exon38 first 90 nt, was cloned into KpnI-XbaI cloning sites of pcDNA 3.1 TOPO vector and named *IKBKAP* skipEx36cal. A piece of WT *IKBKAP* cDNA, containing the last 30 nt of exon1-exon2-exon3 first 110 nt, was cloned into KpnI-XbaI cloning sites of pcDNA 3.1 TOPO vector and named *IKBKAP* fullEx2cal. For *ABL1*, the last 37 nt of exon 2 and first 102 nt of exon 3 were amplified from *ABL1* cDNA, cloned into KpnI-XbaI cloning sites of pcDNA 3.1 TOPO vector and named *ABL1* cal. All plasmid calibrators were linearized with XbaI and serially diluted in a solution of Tris 10 mM, EDTA 1 mM pH 8, containing 20 ng/µl of *E. coli* 16S and 23S rRNA (Roche).

### Real-time PCR assay

The PCR reactions were performed in triplicate in a final volume of 25 µl, including 300 nM primers, 200 nM TaqMan® probe, 12.5 µl of TaqMan® universal PCR master mix (Applied Biosystems) and 5 µl of either cDNA or plasmid calibrator in a AB Prism 7900 HT thermocycler with 50 cycles and the protocol recommended by the manufacturer. For relative quantification and microarray results validation, we selected primer sets and probes, matching sequences present in the IMAGE human cDNA clones of the nylon microarrays with those displayed on the web portal of Applied Biosystems. The assay IDs were the following: Hs00375306_m1 (*PMEPA1*) and Hs00293488_m1 (*S100A16*) for the dysregulated genes in FD, and Hs01003267_m1 (*HPRT1*) and Hs00293488_m1 (*RPLP0*) for reference genes used to normalize the data. We also used previously validated primers and probe for *ABL1* as a third reference gene [62]. Results were calculated using the 2(−ΔΔC_T_) method [63]. For absolute quantification, *IKBKAP* primers and hydrolysis probes (FAM TAMRA) were designed using the Primer 3 software and are listed in Table 2. Serial dilutions of plasmid calibrators (10^6^, 10^5^, 10^4^, 10^3^, 10^2^ copies in 5 µl) were prepared and used to construct the standard curves. The number of *IKBKAP* and *ABL1* transcripts was extrapolated automatically by the Sequence Detection System v2.2.2 software (Applied Biosystems).

### Microarray analysis and normalization

RNA integrity was assessed using an Agilent 2100 Bioanalyser (Palo Alto, CA). Samples with an RNA integrity number (RIN)<9 were excluded from the analysis (the samples concerned were C2P5, C3P5, and FD2P5). Gene expression analyses were carried out with cDNA Nylon microarrays containing 8,780 spotted cDNA clones and radioactive detection as previously described [64], with 5 µg of RNA reverse transcribed (oligo-dT priming) in presence of [α-^33^P]dCTP (Amersham Pharmacia Biotech). Details about microarray construction, clones list, probes preparations, hybridizations and washes have been previously described [65]. After image acquisition, signal intensities were quantified using BZScan software (http://tagc.univ-mrs.fr/bioinformatics/bzscan, [66]). A specific R library that uses the ‘S4’ system of formal classes and methods was used to process and normalize nylon microarray data [67]. Quantile normalization was applied to vector probe data (V) and complex probe data (C), to correct for global intensity and dispersion. Correction by the vector signal was made for each spot signal by calculating a C∶V ratio before log transformation (base 2). No background correction or overshining correction was used. All data are MIAME compliant and have been loaded into ArrayExpress database (http://www.ebi.ac.uk/microarray-as/ae/) under accession number E-MTAB-281.

### Statistical and gene ontology analysis

Significant Analysis of Microarray (SAM version 1.13; Standford University) was applied to determine significant differential gene expression using the Multiexperiment viewer (MEV) program. The data were analyzed using a two-class unpaired response type, which compared control samples versus FD samples. SAM calculated a significant score for each gene based on the gene expression change relative to the standard deviation of repeated values for that gene. We used 100 permutations and a false discovery rate (FDR) of 3%. A total of 50 genes appearing in the heat map generation were called as significant with a p-value<0.006. For gene ontology analysis, we generated a set of human protein associated with the gene appearing as significant with the SAM test, by using the BioKnowledge® Library (BKL) Retriever™ search tool (http://www.biobase-international.com/). This set of proteins was analyzed for overrepresentation of Gene Ontology (GO) Biological Process (BP) terms.

### Boyden chamber-based cell migration assay

hOE-MSCs were detached by trypsin/EDTA, counted and seeded into the upper chamber of transwell polyethylene terephtalate filter membranes with 8 µM diameter pores (BD Biosciences), at a density of 3×10^4^ cells/well, in a final volume of 200 µl serum or serum free culture medium, with or without 100 µM kinetin. Cells were allowed to migrate through the membrane filter for 24h at 37°C, 5% CO_2_. Cells migrating thought the membrane pore and invading the underside surface of the membrane were fixed with 4% paraformaldehyde. Non-migratory cells on the upper membrane surface were removed with a cotton swab, and nuclei were stained with 0.5 µg/mL DNA intercalant Hoechst #33258. For quantitative assessment, the number of stained migrating cells was counted with imageJ software on 10 random fields per membrane filter at ×20 magnification.

## Supporting Information

Figure S1 **50 genes are differentially expressed between control and FD hOE-MSCs.** Heatmap representation of overexpressed (red) and underexpressed (green) genes in 5 controls and 4 FD OE-MSCs at passage 1,2,5, and 9. Normalized signal intensities were treated with the SAM software to highlight the most differentially expressed genes, with a FDR set at 3%.
(TIF)

Figure S2 ***IKBKAP*** **exon 36 inclusion increases after cycloheximide treatment.** NMD pathway was blocked by the translation inhibitor cycloheximide and results in an elevated expression of exon 36-including transcripts in 2 FD OE-MSC cultures (FD3 and FD4) as determined by absolute RT-qPCR. (* *P*<0.05).
(TIF)

Table S1 **Dysregulated genes involved in other processes.**
(DOC)

## References

[1] Axelrod FB, Iyer K, Fish I, Pearson J, Sein ME (1981). Progressive sensory loss in familial dysautonomia.. Pediatrics.

[2] Pearson J, Pytel BA, Grover-Johnson N, Axelrod F, Dancis J (1978). Quantitative studies of dorsal root ganglia and neuropathologic observations on spinal cords in familial dysautonomia.. J Neurol Sci.

[3] Pearson J, Pytel BA (1978). Quantitative studies of sympathetic ganglia and spinal cord intermedio-lateral gray columns in familial dysautonomia.. J Neurol Sci.

[4] Axelrod FB (2004). Familial dysautonomia.. Muscle Nerve.

[5] Anderson SL, Coli R, Daly IW, Kichula EA, Rork MJ (2001). Familial dysautonomia is caused by mutations of the IKAP gene.. Am J Hum Genet.

[6] Slaugenhaupt SA, Blumenfeld A, Gill SP, Leyne M, Mull J (2001). Tissue-specific expression of a splicing mutation in the IKBKAP gene causes familial dysautonomia.. Am J Hum Genet.

[7] Dong J, Edelmann L, Bajwa AM, Kornreich R, Desnick RJ (2002). Familial dysautonomia: detection of the IKBKAP IVS20(+6T→C) and R696P mutations and frequencies among Ashkenazi Jews.. Am J Med Genet.

[8] Cuajungco MP, Leyne M, Mull J, Gill SP, Lu W (2003). Tissue-specific reduction in splicing efficiency of IKBKAP due to the major mutation associated with familial dysautonomia.. Am J Hum Genet.

[9] Hawkes NA, Otero G, Winkler GS, Marshall N, Dahmus ME (2002). Purification and characterization of the human elongator complex.. J Biol Chem.

[10] Close P, Hawkes N, Cornez I, Creppe C, Lambert CA (2006). Transcription impairment and cell migration defects in elongator-depleted cells: implication for familial dysautonomia.. Mol Cell.

[11] Creppe C, Malinouskaya L, Volvert ML, Gillard M, Close P (2009). Elongator controls the migration and differentiation of cortical neurons through acetylation of alpha-tubulin.. Cell.

[12] Solinger JA, Paolinelli R, Kloss H, Scorza FB, Marchesi S (2010). The Caenorhabditis elegans Elongator complex regulates neuronal alpha-tubulin acetylation.. PLoS Genet.

[13] Chen C, Tuck S, Bystrom AS (2009). Defects in tRNA modification associated with neurological and developmental dysfunctions in Caenorhabditis elegans elongator mutants.. PLoS Genet.

[14] Huang B, Johansson MJ, Bystrom AS (2005). An early step in wobble uridine tRNA modification requires the Elongator complex.. RNA.

[15] Esberg A, Huang B, Johansson MJ, Bystrom AS (2006). Elevated levels of two tRNA species bypass the requirement for elongator complex in transcription and exocytosis.. Mol Cell.

[16] Rahl PB, Chen CZ, Collins RN (2005). Elp1p, the yeast homolog of the FD disease syndrome protein, negatively regulates exocytosis independently of transcriptional elongation.. Mol Cell.

[17] Okada Y, Yamagata K, Hong K, Wakayama T, Zhang Y (2010). A role for the elongator complex in zygotic paternal genome demethylation.. Nature.

[18] Lipardi C, Paterson BM (2009). Identification of an RNA-dependent RNA polymerase in Drosophila involved in RNAi and transposon suppression.. Proc Natl Acad Sci U S A.

[19] Chen YT, Hims MM, Shetty RS, Mull J, Liu L (2009). Loss of mouse Ikbkap, a subunit of elongator, leads to transcriptional deficits and embryonic lethality that can be rescued by human IKBKAP.. Mol Cell Biol.

[20] Hims MM, Shetty RS, Pickel J, Mull J, Leyne M (2007). A humanized IKBKAP transgenic mouse models a tissue-specific human splicing defect.. Genomics.

[21] Valensi-Kurtz M, Lefler S, Cohen MA, Aharonowiz M, Cohen-Kupiec R (2010). Enriched population of PNS neurons derived from human embryonic stem cells as a platform for studying peripheral neuropathies.. PLoS One.

[22] Lee G, Papapetrou EP, Kim H, Chambers SM, Tomishima MJ (2009). Modelling pathogenesis and treatment of familial dysautonomia using patient-specific iPSCs.. Nature.

[23] Saha K, Jaenisch R (2009). Technical challenges in using human induced pluripotent stem cells to model disease.. Cell Stem Cell.

[24] Vierbuchen T, Ostermeier A, Pang ZP, Kokubu Y, Sudhof TC (2010). Direct conversion of fibroblasts to functional neurons by defined factors.. Nature.

[25] Kim K, Doi A, Wen B, Ng K, Zhao R (2010). Epigenetic memory in induced pluripotent stem cells.. Nature.

[26] Polo JM, Liu S, Figueroa ME, Kulalert W, Eminli S (2010). Cell type of origin influences the molecular and functional properties of mouse induced pluripotent stem cells.. Nat Biotechnol.

[27] Ghosh Z, Wilson KD, Wu Y, Hu S, Quertermous T (2010). Persistent donor cell gene expression among human induced pluripotent stem cells contributes to differences with human embryonic stem cells.. PLoS One.

[28] Graziadei PP, Graziadei GA (1979). Neurogenesis and neuron regeneration in the olfactory system of mammals. I. Morphological aspects of differentiation and structural organization of the olfactory sensory neurons.. J Neurocytol.

[29] Murrell W, Feron F, Wetzig A, Cameron N, Splatt K (2005). Multipotent stem cells from adult olfactory mucosa.. Dev Dyn.

[30] Delorme B, Nivet E, Gaillard J, Haupl T, Ringe J (2010). The human nose harbors a niche of olfactory ectomesenchymal stem cells displaying neurogenic and osteogenic properties.. Stem Cells Dev.

[31] Feron F, Perry C, Hirning MH, McGrath J, Mackay-Sim A (1999). Altered adhesion, proliferation and death in neural cultures from adults with schizophrenia.. Schizophr Res.

[32] McCurdy RD, Feron F, Perry C, Chant DC, McLean D (2006). Cell cycle alterations in biopsied olfactory neuroepithelium in schizophrenia and bipolar I disorder using cell culture and gene expression analyses.. Schizophr Res.

[33] Murrell W, Wetzig A, Donnellan M, Feron F, Burne T (2008). Olfactory mucosa is a potential source for autologous stem cell therapy for Parkinson's disease.. Stem Cells.

[34] Johansen LD, Naumanen T, Knudsen A, Westerlund N, Gromova I (2008). IKAP localizes to membrane ruffles with filamin A and regulates actin cytoskeleton organization and cell migration.. J Cell Sci.

[35] Slaugenhaupt SA, Mull J, Leyne M, Cuajungco MP, Gill SP (2004). Rescue of a human mRNA splicing defect by the plant cytokinin kinetin.. Hum Mol Genet.

[36] Hims MM, Ibrahim EC, Leyne M, Mull J, Liu L (2007). Therapeutic potential and mechanism of kinetin as a treatment for the human splicing disease familial dysautonomia.. J Mol Med.

[37] Gold-von Simson G, Goldberg JD, Rolnitzky LM, Mull J, Leyne M (2009). Kinetin in familial dysautonomia carriers: implications for a new therapeutic strategy targeting mRNA splicing.. Pediatr Res.

[38] Tanabe S, Sato Y, Suzuki T, Suzuki K, Nagao T (2008). Gene expression profiling of human mesenchymal stem cells for identification of novel markers in early- and late-stage cell culture.. J Biochem.

[39] Wagner W, Horn P, Castoldi M, Diehlmann A, Bork S (2008). Replicative senescence of mesenchymal stem cells: a continuous and organized process.. PLoS One.

[40] Cheishvili D, Maayan C, Smith Y, Ast G, Razin A (2007). IKAP/hELP1 deficiency in the cerebrum of familial dysautonomia patients results in down regulation of genes involved in oligodendrocyte differentiation and in myelination.. Hum Mol Genet.

[41] Zhang X, Klueber KM, Guo Z, Cai J, Lu C (2006). Induction of neuronal differentiation of adult human olfactory neuroepithelial-derived progenitors.. Brain Res.

[42] Wolozin B, Sunderland T, Zheng BB, Resau J, Dufy B (1992). Continuous culture of neuronal cells from adult human olfactory epithelium.. J Mol Neurosci.

[43] Roisen FJ, Klueber KM, Lu CL, Hatcher LM, Dozier A (2001). Adult human olfactory stem cells.. Brain Res.

[44] Zhang X, Klueber KM, Guo Z, Lu C, Roisen FJ (2004). Adult human olfactory neural progenitors cultured in defined medium.. Exp Neurol.

[45] Winstead W, Marshall CT, Lu CL, Klueber KM, Roisen FJ (2005). Endoscopic biopsy of human olfactory epithelium as a source of progenitor cells.. Am J Rhinol.

[46] Feron F, Perry C, McGrath JJ, Mackay-Sim A (1998). New techniques for biopsy and culture of human olfactory epithelial neurons.. Arch Otolaryngol Head Neck Surg.

[47] Othman M, Lu C, Klueber K, Winstead W, Roisen F (2005). Clonal analysis of adult human olfactory neurosphere forming cells.. Biotech Histochem.

[48] Viegas MH, Gehring NH, Breit S, Hentze MW, Kulozik AE (2007). The abundance of RNPS1, a protein component of the exon junction complex, can determine the variability in efficiency of the Nonsense Mediated Decay pathway.. Nucleic Acids Res.

[49] Bateman JF, Freddi S, Nattrass G, Savarirayan R (2003). Tissue-specific RNA surveillance? Nonsense-mediated mRNA decay causes collagen X haploinsufficiency in Schmid metaphyseal chondrodysplasia cartilage.. Hum Mol Genet.

[50] Resta N, Susca FC, Di Giacomo MC, Stella A, Bukvic N (2006). A homozygous frameshift mutation in the ESCO2 gene: evidence of intertissue and interindividual variation in Nmd efficiency.. J Cell Physiol.

[51] Holmberg C, Katz S, Lerdrup M, Herdegen T, Jaattela M (2002). A novel specific role for I kappa B kinase complex-associated protein in cytosolic stress signaling.. J Biol Chem.

[52] Kim JH, Lane WS, Reinberg D (2002). Human Elongator facilitates RNA polymerase II transcription through chromatin.. Proc Natl Acad Sci U S A.

[53] Cornez I, Creppe C, Gillard M, Hennuy B, Chapelle JP (2008). Deregulated expression of pro-survival and pro-apoptotic p53-dependent genes upon Elongator deficiency in colon cancer cells.. Biochem Pharmacol.

[54] Watanabe Y, Itoh S, Goto T, Ohnishi E, Inamitsu M (2010). TMEPAI, a transmembrane TGF-beta-inducible protein, sequesters Smad proteins from active participation in TGF-beta signaling.. Mol Cell.

[55] Stamm S, Riethoven JJ, Le Texier V, Gopalakrishnan C, Kumanduri V (2006). ASD: a bioinformatics resource on alternative splicing.. Nucleic Acids Res.

[56] Cohen L, Henzel WJ, Baeuerle PA (1998). IKAP is a scaffold protein of the IkappaB kinase complex.. Nature.

[57] Pros E, Fernandez-Rodriguez J, Benito L, Ravella A, Capella G (2009). Modulation of aberrant NF1 pre-mRNA splicing by kinetin treatment.. Eur J Hum Genet.

[58] Viktorov IV, Savchenko EA, Chekhonin VP (2007). Spontaneous neural differentiation of stem cells in culture of human olfactory epithelium.. Bull Exp Biol Med.

[59] Carmel I, Tal S, Vig I, Ast G (2004). Comparative analysis detects dependencies among the 5′ splice-site positions.. RNA.

[60] Ibrahim EC, Hims MM, Shomron N, Burge CB, Slaugenhaupt SA (2007). Weak definition of IKBKAP exon 20 leads to aberrant splicing in familial dysautonomia.. Hum Mutat.

[61] Ule J, Ule A, Spencer J, Williams A, Hu JS (2005). Nova regulates brain-specific splicing to shape the synapse.. Nat Genet.

[62] Beillard E, Pallisgaard N, van der Velden VH, Bi W, Dee R (2003). Evaluation of candidate control genes for diagnosis and residual disease detection in leukemic patients using ‘real-time’ quantitative reverse-transcriptase polymerase chain reaction (RQ-PCR) - a Europe against cancer program.. Leukemia.

[63] Livak KJ, Schmittgen TD (2001). Analysis of relative gene expression data using real-time quantitative PCR and the 2(−Delta Delta C(T)) Method.. Methods.

[64] Ballester B, Ramuz O, Gisselbrecht C, Doucet G, Loi L (2006). Gene expression profiling identifies molecular subgroups among nodal peripheral T-cell lymphomas.. Oncogene.

[65] Talby L, Chambost H, Roubaud MC, N'Guyen C, Milili M (2006). The chemosensitivity to therapy of childhood early B acute lymphoblastic leukemia could be determined by the combined expression of CD34, SPI-B and BCR genes.. Leuk Res.

[66] Lopez F, Rougemont J, Loriod B, Bourgeois A, Loi L (2004). Feature extraction and signal processing for nylon DNA microarrays.. BMC Genomics.

[67] Gentleman RC, Carey VJ, Bates DM, Bolstad B, Dettling M (2004). Bioconductor: open software development for computational biology and bioinformatics.. Genome Biol.

