# Supplementary figures and images for: Olfactory Stem Cells, a New Cellular Model for Studying Molecular Mechanisms Underlying Familial Dysautonomia
Source: PLoS One. 2010 Dec 20;5(12):e15590. doi: 10.1371/journal.pone.0015590 (PMC3004942; doi:10.1371/journal.pone.0015590)

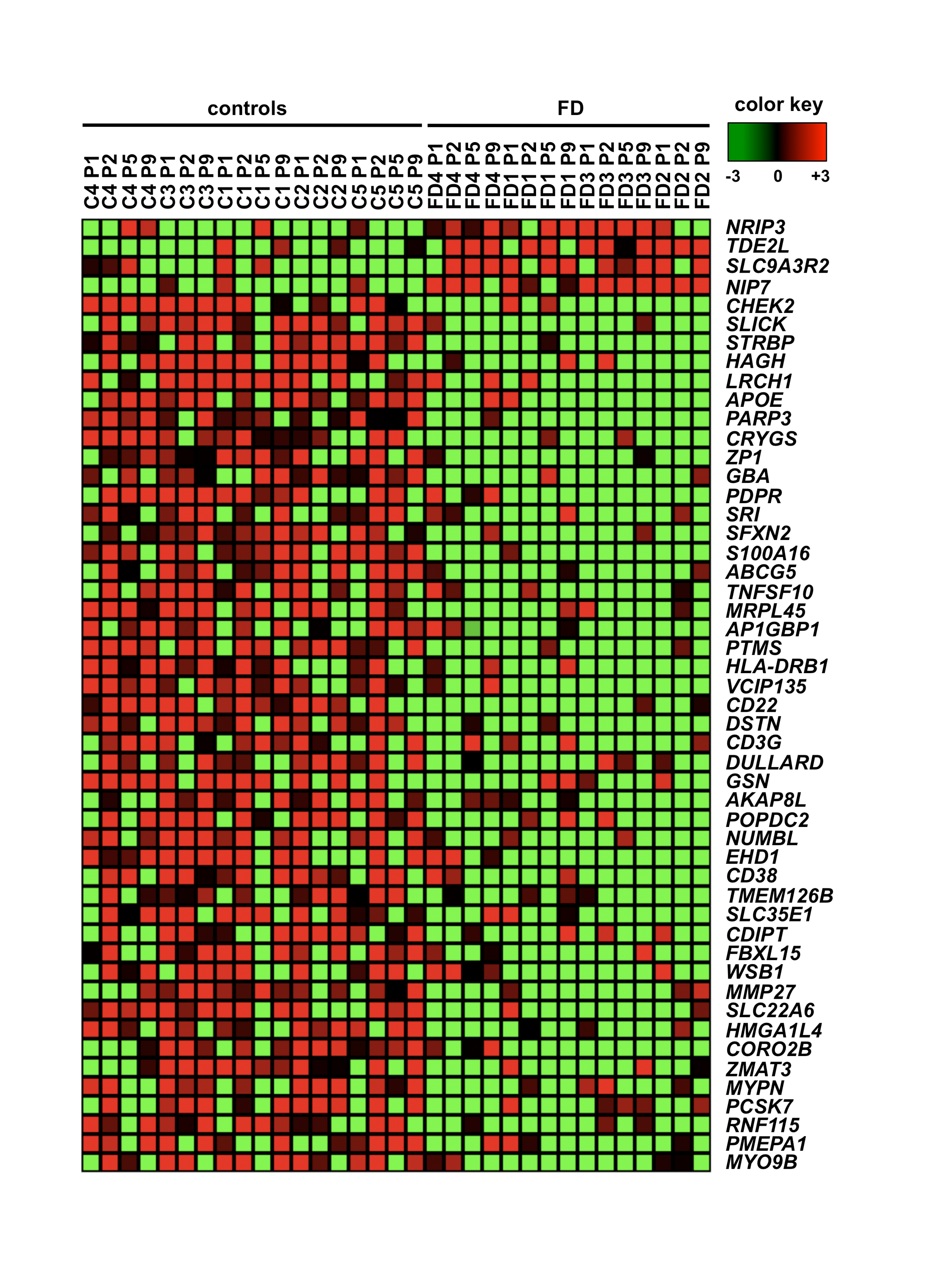

Supplement: Figure S1 — 50 genes are differentially expressed between control and FD hOE-MSCs. Heatmap representation of overexpressed (red) and underexpressed (green) genes in 5 controls and 4 FD OE-MSCs at passage 1,2,5, and 9. Normalized signal intensities were treated with the SAM software to highlight the most differentially expressed genes, with a FDR set at 3%. (TIF) [file pone.0015590.s001.tif]

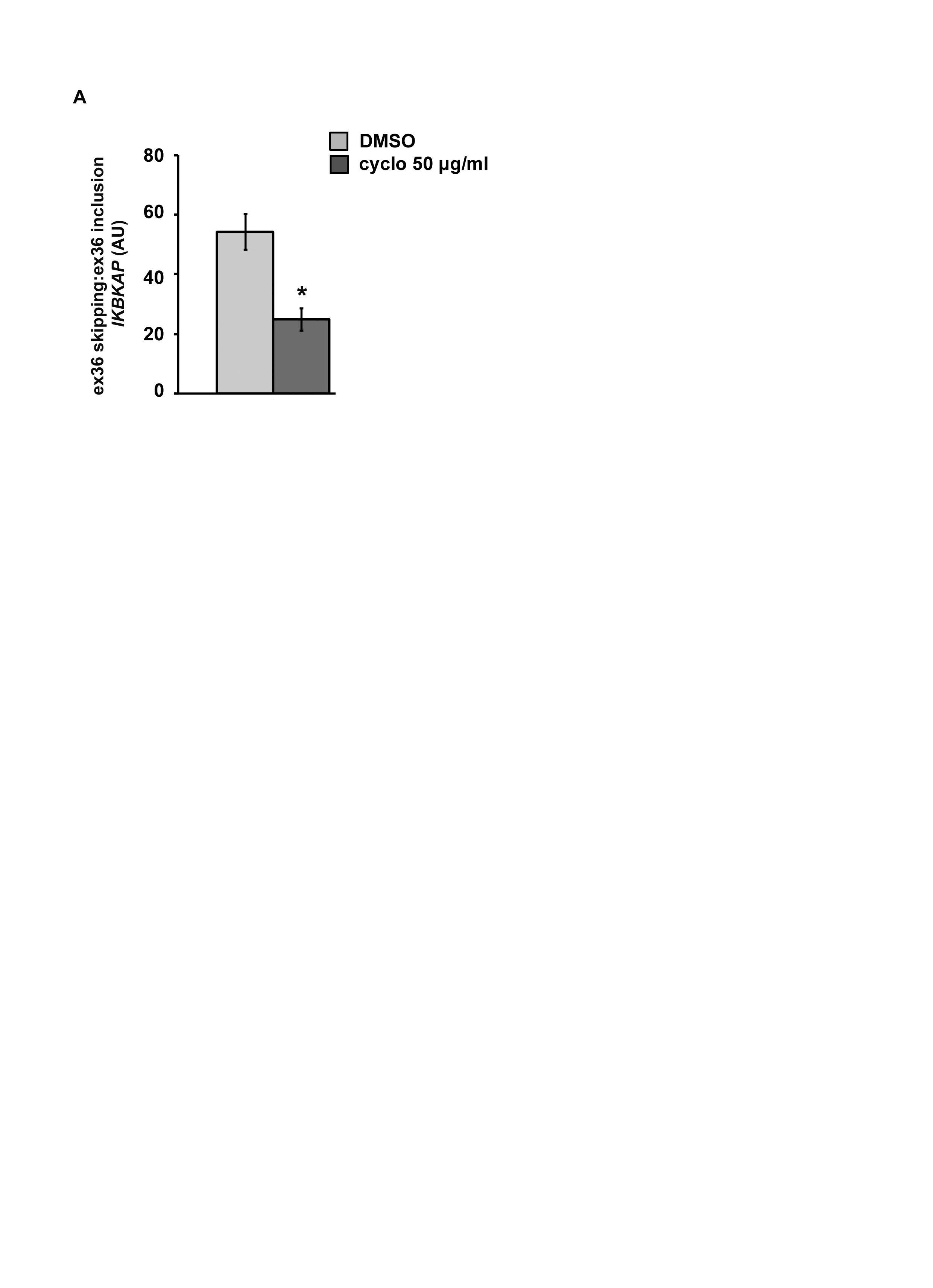

Supplement: Figure S2 — IKBKAP exon 36 inclusion increases after cycloheximide treatment. NMD pathway was blocked by the translation inhibitor cycloheximide and results in an elevated expression of exon 36-including transcripts in 2 FD OE-MSC cultures (FD3 and FD4) as determined by absolute RT-qPCR. (* P<0.05). (TIF) [file pone.0015590.s002.tif]
