# Supplementary material for: Olfactory Stem Cells, a New Cellular Model for Studying Molecular Mechanisms Underlying Familial Dysautonomia
Source: PLoS One. 2010 Dec 20;5(12):e15590. doi: 10.1371/journal.pone.0015590 (PMC3004942; doi:10.1371/journal.pone.0015590)
Supplement: Table S1 — Dysregulated genes involved in other processes. (DOC) [file pone.0015590.s003.doc]

| **Gene** | Clone ID | **FC** | ***p*-value** | Biological process | **Studies** |
| --- | --- | --- | --- | --- | --- |
| *POPDC2* | 487912 | -2,29 | 0,00403 | putative role in heart development |  |
| *HAGH* | 341240 | -2,22 | 0,00019 | regulation of glutathione of pyruvate metabolism process |  |
| *MRPL45* | 297955 | -1,85 | 0,00074 | translation of mitochondrial genome |  |
| *SYNRG* | 2345174 | -1,82 | 0,00111 | positive regulation of transferrin receptor recycling |  |
| *PDPR* | 240361 | -1,77 | 0,00059 | insulin-induced activation of mitochondrial PDP in adipose tissue |  |
| *VCPIP1* | 240722 | -1,75 | 0,00068 | post mitotic Golgi apparatus reassembly |  |
| *SRI* | 245759 | -1,70 | 0,00037 | cellular calcium ion homeostasis - heart development |  |
| *CD3G* | 110938 | -1,70 | 0,00184 | T cell developement - cell adhesion |  |
| *CRYGS* | 229984 | -1,68 | 0,00020 | cell morphogenesis - visual perception |  |
| *HLA-DRB1* | 240655 | -1,66 | 0,00058 | presentation of endegenous peptide antigen | *2 |
| *TMEM126B* | 269998 | -1,64 | 0,00098 | uncharacterized hypothalamic protein - unknown role |  |
| *ZP1* | 757505 | -1,60 | 0,00014 | single fertilization |  |
| *AKAP8L* | 171679 | -1,58 | 0,00051 | regulation of DNA replication initiation by chromatin condensation |  |
| *EHD1* | 745019 | -1,58 | 0,00081 | endocytosis - IGF1- mediated signaling repression |  |
| *PTMS* | 490340 | -1,54 | 0,00015 | regulation of glucocorticoid dependent gene transcription | *1 |
| *NRIP3* | 728684 | 1,61 | 0,00005 | regulation of transcription |  |
| *NIP7* | 376672 | 1,83 | 0,00004 | ribosome biogenesis | *1 |

*1 = Lee et al. 2009

*2 = Close et al. 2006
